# MEG-Based Detection of Voluntary Eye Fixations Used to Control a Computer

**DOI:** 10.3389/fnins.2021.619591

**Published:** 2021-02-05

**Authors:** Anastasia O. Ovchinnikova, Anatoly N. Vasilyev, Ivan P. Zubarev, Bogdan L. Kozyrskiy, Sergei L. Shishkin

**Affiliations:** ^1^MEG Center, Moscow State University of Psychology and Education, Moscow, Russia; ^2^Laboratory for Neurocognitive Technologies, NRC Kurchatov Institute, Moscow, Russia; ^3^Department of Physics of Extreme States of Matter, National Research Nuclear University MEPhI, Moscow, Russia; ^4^Laboratory for Neurophysiology and Neuro-Computer Interfaces, M. V. Lomonosov Moscow State University, Moscow, Russia; ^5^Department of Neuroscience and Biomedical Engineering, Aalto University School of Science, Espoo, Finland; ^6^Department of Data Science, EURECOM, Biot, France

**Keywords:** MEG, brain-computer interface, hybrid brain-computer interface, gaze-based interaction, convolutional neural network, classification, intention

## Abstract

Gaze-based input is an efficient way of hand-free human-computer interaction. However, it suffers from the inability of gaze-based interfaces to discriminate voluntary and spontaneous gaze behaviors, which are overtly similar. Here, we demonstrate that voluntary eye fixations can be discriminated from spontaneous ones using short segments of magnetoencephalography (MEG) data measured immediately after the fixation onset. Recently proposed convolutional neural networks (CNNs), linear finite impulse response filters CNN (LF-CNN) and vector autoregressive CNN (VAR-CNN), were applied for binary classification of the MEG signals related to spontaneous and voluntary eye fixations collected in healthy participants (*n* = 25) who performed a game-like task by fixating on targets voluntarily for 500 ms or longer. Voluntary fixations were identified as those followed by a fixation in a special confirmatory area. Spontaneous vs. voluntary fixation-related single-trial 700 ms MEG segments were non-randomly classified in the majority of participants, with the group average cross-validated ROC AUC of 0.66 ± 0.07 for LF-CNN and 0.67 ± 0.07 for VAR-CNN (*M* ± SD). When the time interval, from which the MEG data were taken, was extended beyond the onset of the visual feedback, the group average classification performance increased up to 0.91. Analysis of spatial patterns contributing to classification did not reveal signs of significant eye movement impact on the classification results. We conclude that the classification of MEG signals has a certain potential to support gaze-based interfaces by avoiding false responses to spontaneous eye fixations on a single-trial basis. Current results for intention detection prior to gaze-based interface’s feedback, however, are not sufficient for online single-trial eye fixation classification using MEG data alone, and further work is needed to find out if it could be used in practical applications.

## Introduction

Brain-computer interfaces (BCIs) are a promising tool that could augment human-computer interaction for patients with motor disabilities and even for healthy users (Allison et al., 2007; Nijholt et al., 2008; Blankertz et al., 2016; Cinel et al., 2019). Moreover, fluent, direct translation of human intentions into actions are often expected from the BCI technology in the future (Martins et al., 2019). However, the performance of the existing noninvasive BCIs is too low, while the invasive BCIs are associated with high risk and cost.

Gaze-based systems using eye-tracking technology provide an intuitive way to control the cursor position and interact with elements of graphical user interface using intentional eye dwells. Whenever dwell duration exceeds a pre-defined threshold, the system interprets the dwell as a command similar to a mouse click. However, there is a trade-off between fluent interaction and error rate: while long dwell time thresholds make the interaction tiresome, short thresholds provide a notably effortless interaction but also lead to frequent misclassification of spontaneous dwells as intended ones (Jacob, 1990). Such false positives are remarkably difficult to avoid because eye movements serve primarily for vision and easily escape conscious control (Jacob, 1990). To solve this problem, Ihme and Zander (2011) and Protzak et al. (2013) proposed to use a passive BCI (Zander and Kothe, 2011), which could detect the expectation-related brain activity measured by the EEG in eye fixations. They argued that this activity could be indicative of the intentional use of the fixation, because in this case the user is aware of the imminent interface feedback. Spontaneous fixations, in contrast, are being made without expectation of the interface triggering, and therefore are not accompanied by such expectation-related EEG activity. Thus, a passive BCI may increase the performance of gaze-based interfaces by preventing responses to unintentional eye dwells (Ihme and Zander, 2011; Protzak et al., 2013). In our previous work, using EEG, we applied this approach to a realistic gaze interaction model implemented as a gaze-controlled game (Shishkin et al., 2016; Nuzhdin et al., 2017).

To make feasible such interaction, the eye-brain-computer interface (EBCI) should provide high on-the-fly classification performance using short segments of single-trial data. In our experiments with an online EBCI that classified gaze fixations using 300 ms long EEG segments non-random classification was demonstrated; however, online classification performance was low due to a high false-positive rate of the BCI classifier (Nuzhdin et al., 2017). One explanation of the low performance could be insufficient spatial resolution of the EEG or its sub-optimal sensitivity to underlying neural sources of interest. To address this possibility, in this study we focused on measuring the magnetic component of the brain activity in a similar task using MEG, which provides higher spatial resolution and sensitivity to sources with orientation not seen in the EEG.

We hypothesized that brain signals accompanying a voluntary attempt to select a screen object with gaze and spontaneous gaze behaviors, such as used for visual exploration, should be different not only due to the presence of components related to the feedback expectation in the case of voluntary control but also because this control should involve distinct brain activity. Voluntary gaze behavior has long been studied (Hallett, 1978; Ettinger et al., 2008), however, the experimental tasks used in such studies (e.g., anti-saccades, delayed response task, etc.) were very demanding compared to the easily executed, relatively short gaze fixations that can be used for gaze-based control. To our knowledge, no published works on classification of MEG activity related to gaze-based control have been undertaken so far. MEG use for practical BCI application has begun to be considered only recently, with the advances in Optically-Pumped Magnetometers (OPMs) development (Paek et al., 2020).

Until recently, the MEG has not been considered a practical measurement technique for BCI applications due to its low portability and high cost. Recent developments in MEG sensor technology, however, hold great promise to change that in the near future. Particularly, OPMs do not require expensive liquid helium to operate, making the system portable and less expensive while providing superior spatial resolution (Boto et al., 2016, 2017, 2018, 2019, 2020; Borna et al., 2017, 2020; Iivanainen et al., 2017, 2019; Hill et al., 2019, 2020; Limes et al., 2020; Zhang R. et al., 2020; Zhang X. et al., 2020). Compared to conventional MEG, OPM-based systems are also more motion-robust (Boto et al., 2018; Hill et al., 2019). Note that all types of MEG technology have an important practical advantage over the EEG: It does not require the application of electrically conductive gel on the skin or pressing the skin with dry electrodes, which are needed for EEG recording. Taking all these features together, it seems likely that further development of OPM-based MEG systems will make MEG-based BCIs widely applicable. First attempts to build a MEG-BCI system based on the OPM technology are already being made (Paek et al., 2020). Further decrease in price of OPM sensors seems highly likely, thus enhancement of the gaze-based interaction with OPM-MEG may also become affordable.

Due to the similarity of MEG and EEG signals, it seems plausible to assume that the MEG classification task is associated with the same difficulties as for the EEG: Low signal-to-noise ratio (SNR); complex, high-dimensional spatiotemporal structure; and insufficient *a priori* knowledge of the informative components. Deep learning approaches were recently adopted in the field of developing EEG-based BCIs to deal with these complex issues. CNNs employing these approaches in form of shallow network architectures enabled better classification performance compared to the classifiers traditionally used in EEG-based BCIs (Lawhern et al., 2016, 2018; Schirrmeister et al., 2017; Lotte et al., 2018). When applied to the eye fixation-related data from our earlier experiments (Shishkin et al., 2016) a compact CNN, the EEGNet (Lawhern et al., 2016, 2018) with optimized hyperparameters provided a 16% improvement over the EEG classification results obtained with the previously used linear classifier (Kozyrskiy et al., 2018). For the MEG signal, CNNs with a different architecture have been proposed, providing an improvement over the EEGNet results (Zubarev et al., 2019). These new networks utilize spatiotemporal structure in the MEG data to extract informative components of the MEG signal. They assume that the MEG measurements are generated by a linear (spatial) mixture of a limited number of latent sources, which evolve non-linearly over time. However, these new CNNs were tested on several typical BCI tasks (Zubarev et al., 2019), based on motor imagery or perception of stimuli, where relatively fast components of the signal were expected to be useful for classification. In our previous work with the EEG-based classification of the voluntary and spontaneous fixations during gaze-based interaction (Shishkin et al., 2016) the informative signal component was a slow deflection. Preliminary analysis of the MEG data indicated that in this case the signal components that discriminated the classes of the voluntary and spontaneous fixations were very slow, such as having the form of a trend developing along most of the epoch of interest, or resembling just a quarter of a full period of a sine wave. Other characteristics of the MEG data in our task and in the BCI tasks tested by Zubarev et al. (2019) also could be different. Thus, it was not possible to predict if these CNNs will be effective for the classification of spontaneous and voluntary gaze fixations.

Thus, in the current study we could not make clear prior predictions, based on the existing literature, of what type of MEG components could differentiate the voluntary eye fixations used to trigger commands from the spontaneous eye fixations. Deep learning-based approaches developed recently for EEG (Lawhern et al., 2016, 2018; Schirrmeister et al., 2017) and MEG (Zubarev et al., 2019) classification, however, do not require prior knowledge about informative features: they combine feature extraction and classification in a single computational framework, allowing to explore features informing the classification.

The objective of this study was to determine if short single-trial segments of MEG data related to voluntary and spontaneous eye fixations in a gaze-based control task can be distinguished. For this purpose, we applied CNNs described in Zubarev et al. (2019) to the MEG data gathered along with spontaneous eye fixations and fixations voluntarily used to trigger a command in an experiment similar to those we used previously to collect similar EEG data (Shishkin et al., 2016; Kozyrskiy et al., 2018).

## Materials and Methods

### Experiment Paradigm and Gaze Data Processing

We used MEG data from a study by our group (will be published elsewhere) recorded at MEG Center (a division of the Moscow State University for Psychology and Education, Moscow, Russia) in 32 healthy volunteers. The study was performed according to Helsinki Declaration and with the approval of the local ethical committee (protocol 12.03.2015/1). The participants played *EyeLines* game, the gaze-controlled version of *Lines*, a turn-based computer board game (also known as *Color Lines –* Color Lines, 2003). This study was designed to identify early MEG markers of intention preceding the eye fixation or appearing early in its course. The previous EEG study (Shishkin et al., 2016) was focused on the expectancy-related EEG component that should be prominent later during the fixation, i.e., closer to the fixation-related feedback, thus the participants’ task included voluntary fixations preceded by a relatively large saccade. Such a saccade could significantly affect the time interval around the voluntary fixation onset, by altering the MEG itself and by producing a strong oculographic artifact. To avoid such feature in the voluntary fixations, the order of eye fixations (dwells) required to make a move in *EyeLines* was made different in the MEG study from one used in the original game version in Shishkin et al. (2016).

**FIGURE 1:**
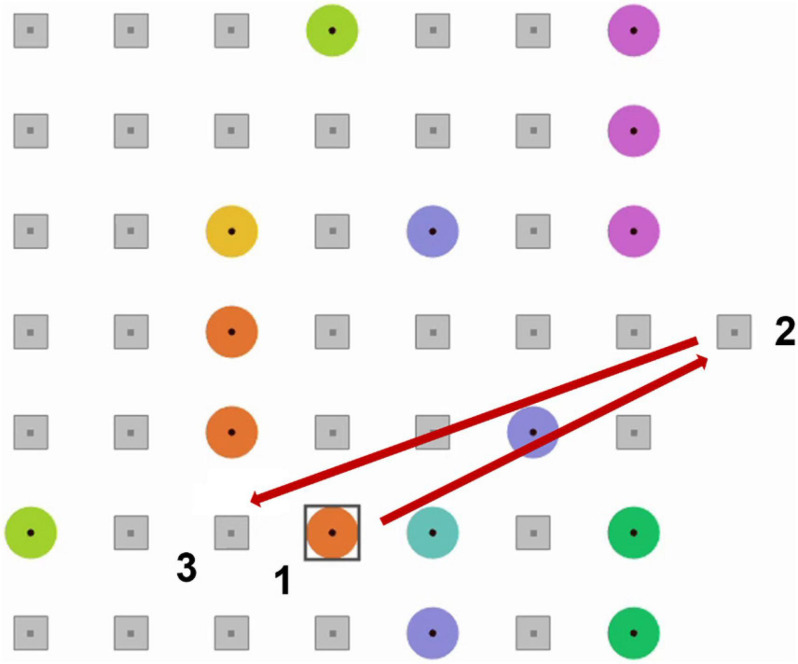
An example of *EyeLine* game board display and an example of a sequence of eye fixations needed to make a move. 1, a target ball; 2, the confirmatory position; 3, a free cell to which the ball will be re-located. A square frame appeared around a ball when it was fixated for more than 500 ms.

In *EyeLines*, like in *Lines*, players make straight lines of colored “balls” by re-locating the balls which are presented at a game board on a screen (Figure 1). To make a move, the player of *EyeLines* first needed to select a ball (“1” in Figure 1) with 500 ms or longer eye fixation on it. After 500 ms of fixating the ball, a preliminary selection was indicated by a square frame around the ball (shown in Figure 1). This could happen not only when the fixation was intentional but also when it was accidental (spontaneous). To make a move, the participant had to confirm the selection by a confirmatory eye fixation on a remote position (“2” in Figure 1), and then on the position to which they wanted to relocate the ball (“3” in Figure 1), each time 500 ms or longer. With this order, a large saccade from the confirmation button preceded not the fixation on the ball but the fixation on the free cell, to which the ball should be moved. The saccades preceding the fixations on the ball were made from the locations inside the game board in the case of both spontaneous and voluntary fixations. Thus, these saccades had, on average, much smaller amplitude than the saccades from the game board, and did not differ between the spontaneous and voluntary classes. To support the detection of the user’s intention in the context of gaze-based interaction, the BCI must make the decision based on the MEG data collected before the time where the feedback is normally given in the case that the BCI is not used. The large saccade to the confirmation position appeared only after the feedback to the eye dwell was given (in our case, at about 500 ms from the fixation onset), plus some reaction time (typically around 300 ms), so the data collected before 500 ms from the fixation onset could be safely used for classification.

Experiment protocol mainly followed the EEG study by Shishkin et al. (2016) (except for 1000-ms dwell time threshold, which was not used in the current study). After a practice, a participant played eight 5-min games in total. The game board (Figure 1) was centered on the screen. The remote position for selection confirmation was located outside the board, on its left (in half of the games) or right side (in another half of the games). This left/right alignment alternated in the subsequent games. The left-right-left- right-… vs. right-left-right-left-… order of games with different confirmation position alignment was assigned to the participants randomly. MEG-compatible *EyeLink 1000 Plus* eye tracker (*SR Research, Canada*) provided gaze coordinates in real time at 500 Hz for the first half of recordings and at 1000 Hz for the second half (sampling rate was different for technical reasons). In the offline analysis, gaze coordinates were upsampled to 1000 Hz for the first half of the recordings. Fixations were detected by an online spatial dispersion-based algorithm that triggered game events (Shishkin et al., 2016).

In the current study, we considered voluntary fixations to be only 500 ms or longer eye dwells on a ball (further referred as “ball fixations” for brevity) that were terminated by a saccade within 500 ms after ball-selection feedback onset which was followed by a fixation on the confirmation button no later than 200 ms after the feedback. Additionally, both voluntary and spontaneous dwells had to display a clear initial fixation onset (as defined by the default *EyeLink* algorithm) within 50 ms of the dwell onset (Note that the detection criterion allowed for small saccades during the dwell time period, so that, strictly speaking, this period often comprised two or more fixations. However, in this paper we mainly use the term “fixation” for the whole dwell duration, as it is typically used in the gaze interaction literature). Ball selection followed by a delayed confirmation were not included into the analysis, because at least some of them could correspond to cases when a participant made a decision only after seeing the feedback, while the fixation was actually spontaneous. In contrast, fast saccade to the confirmatory position could be made only if such confirmation was already planned during the fixation, so we could be sure that the fixations quickly followed by a confirmation were intentional.

Ball fixations not followed by a confirmatory fixation (i.e., followed by other ball fixations or fixations on other balls that did not exceed the dwell time threshold) were considered as spontaneous fixations.

Additional criteria were applied to exclude fixations with minor issues related to specific strategies of the participants, eye tracking errors, etc. After applying these criteria, seven participants were excluded due to the low number of voluntary trials (less than 70), thus we further used only the MEG recordings from 25 participants. More specific details about the experiment and the data will be published elsewhere.

### MEG Data Processing and Features Extraction

Magnetoencephalography was recorded at 1000 Hz sampling rate using an *Elekta Neuromag Vectorview* (*MEGIN/Elekta Oy*, Helsinki, Finland) MEG system, with 306 sensors at 102 positions around the head (two orthogonal planar gradiometers and a magnetometer per position). Sensors with contaminated signals were manually rejected, then *MaxFilter* routine (*MEGIN/Elekta Oy*, Helsinki, Finland) was applied with the following settings: Temporal Signal Space Separation (tSSS) (Taulu and Simola, 2006) with *t* = 10 s and *corr* = 0.9, movement correction (MaxMove) and normalizing head origin to the standard position (more details can be found in *MaxFilter User’s Guide*, The Ohba Analysis Group, 2010).

The total trial count, per participant, was (*M* ± SD) 128 ± 38 for voluntary and 294 ± 77 for spontaneous fixations. To balance the number of trials between the classes, randomly selected trials were removed from the set of spontaneous trials (which was initially larger than the set of voluntary trials in all participants) until its size became equal to the size of the voluntary trial set. After class balancing, it became 128 ± 38 trials per class.

Signals from the planar gradiometers (204 channels) were lowpass filtered below 45 Hz and normalized by subtracting the trial mean calculated in each channel separately and dividing by trial standard deviation calculated for data collapsed over channels. Data from magnetometers were not used because of lower spatial resolution and SNR (signal-to-noise ratio) due to omnidirectional signal pickup pattern, compared to gradiometers (in particular, the data from magnetometers are more sensitive to ocular artifacts). Signals were downsampled to 125 Hz to reduce their dimensionality and expand the CNN receptive field in the time domain (Luo et al., 2017). The above procedure resulted in 204 × 87 data dimension (channels × down-sampled timepoints) in the case of −0.2 … 0.5 s features per trial.

Preprocessed MEG epochs of −0.2 … 0.5 s related to the onset of the fixation were used as input to CNN in the main analysis, where we tried to model real-time use of the MEG-based BCI, which has to classify the fixation-related data immediately after the dwell time threshold (0.5 s) is exceeded. The left border was placed at −0.2 s, because, as our preliminary analysis showed, starting the time interval from −0.4 s provided no improvement, and further extension of the interval to the left could lead to incorporating remnants of previous gaze behavior and the reaction to the feedback to an earlier eye dwell on a ball. An additional analysis using longer MEG epochs from −0.2 … 1.0 s interval was performed to check that the classifier was operating correctly. Unlike in the main analysis, this longer time interval also included a response to the presentation of the visual feedback (a square frame appearing around the gazed ball approximately at 0.5 s). Although the feedback did not differ between voluntary and spontaneous fixations, it likely could lead to significantly different MEG response, in particular in the time range of the P300 wave. Moreover, gaze behavior following the feedback clearly differed between voluntary and spontaneous fixations, so MEG signal could include strongly different patterns of eye movement artifacts. Thus, we expected that the data from the −0.2 … 1.0 s interval could be easily classified, and the classification result would provide an estimate of the CNN architectures’ capability to discriminate single-trial fixation-related MEG data in an “easy classification” scenario.

### Convolutional Neural Networks

**FIGURE 2:**
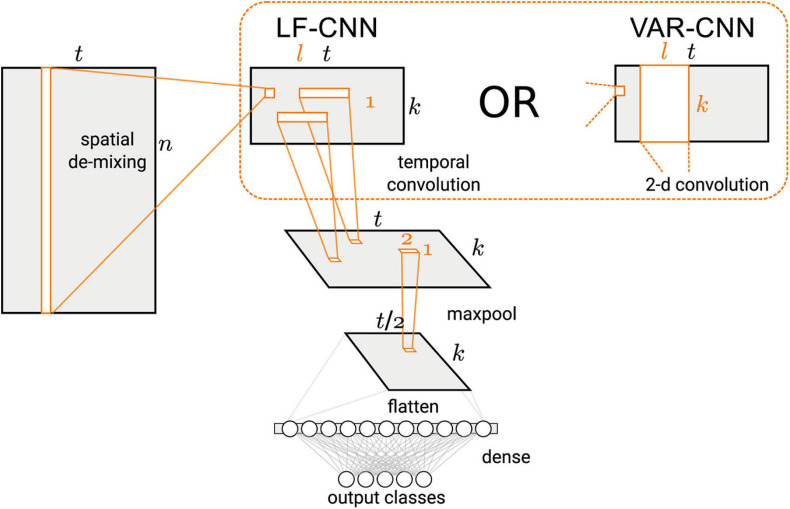
Architecture of the CNNs used in this study (Figure 1 in Zubarev et al., 2019, license CC BY-NC-ND 4.0).

Binary classification of the MEG signals corresponding to spontaneous and voluntary gaze fixations was performed using two CNNs, VAR-CNN and LF-CNN (for architectures details see Figure 2 and Zubarev et al., 2019). These models consist of two convolutional layers: the first one is fully connected spatial convolution, identical for both models; the second layer corresponds to temporal (in case of LF-CNN) or spatiotemporal convolution (in case VAR-CNN). The LF-CNN model is based on assumption that time courses from different channels do not interact and have unique spectral fingerprints. It can be considered as applying linear finite-impulse-response filters (hence LF) that specifically capture the fingerprint of each spatial component. VAR-CNN allows estimating the interactions between the spatial components and can be considered as a vector autoregressive model (VAR-CNN) of the component time courses. CNNs were implemented by AO using Keras library following the network description in Zubarev et al. (2019).

The CNNs architecture comprised a spatial linear convolution layer followed by a temporal convolution layer with rectified linear units (ReLU) non-linearity and max-pooling over 4 time adjacent samples. Two variants were proposed for the temporal layer: separable temporal convolution, applied to the time courses of the latent sources (LSs), was utilized by LF-CNN modification, whereas a spatiotemporal convolution, assuming the interactions between the spatial components, was used in VAR-CNN modification. The classification step comprised a single fully-connected layer followed by a softmax normalization.

### Hyperparameter Setting

First, we attempted to tune several architecture parameters, namely the number of LSs, the length of the temporal filter and max-pooling parameters by means of *hyperopt* Python package^1^ and a grid search. Unfortunately, neither approach resulted in a significant increase of the classifier performance estimated as the ROC AUC values (area under the curve of the receiver operating characteristic). Therefore, we set the number of LSs and the length of the temporal filter to arbitrary values chosen low enough to avoid overfitting, to save the flexibility of convolutions and to decrease the training time. Specifically, for both types of CNN the number of LSs was set to 16, whereas the length of time filters was chosen to be 14, which was equal to 100 ms.

A combination of drop-out (Srivastava et al., 2014) applied to the output convolutional layer and l_1_-penalty applied to weights of the convolutional layers were utilized as a regularization strategy. As a loss function, binary cross-entropy was utilized. We used the Adam optimization algorithm with a batch size of 100 and learning rate of 3 × 10^–4^ to optimize binary cross-entropy between model predictions and true labels.

### LF-CNN Parameters Interpretation

The LF model was supposed to allow the interpretation of the model parameters in terms of the underlying neural activity, since the model structure reflected the assumptions about the data generation process (Zubarev et al., 2019).

According to the underlying generative model, the observed data *X* was considered as a function of some latent (that is, hidden) variables called components or LSs (Haufe et al., 2014). In the linear case such a model can be written as,

(1) X=A⁢S+ε,

where *X* ∈ *ℝ*^*n*×*t*^ is a single MEG epoch, *n* is the number of channels (e.g., *n=204* in case of gradiometers), *S* ∈ *ℝ*^*k*×*t*^ is the representation of a MEG epoch in space of *k* underlying LSs, and ε is an additive Gaussian white observation noise.

Since LF-CNN is an example of a discriminative (as opposed to generative) model, we followed a procedure suggested by Haufe et al. (2014) to obtain activation patterns from the spatial filters *W* trained by the spatial convolution layer (see Figure 2). These filters are related to the spatial activation patterns *A* of the LSs in the generative model (eq. 1) via,

(2) $$W^{T}\,X=\hat{S},$$

(3) $$A=\Sigma_{X}\,W\,\Sigma_{\hat{S}}^{-1},$$

where *Σ*_*X*_ is the spatial data covariance and $\Sigma_{\hat{S}}^{-1}$ is the precision matrix of the latent time courses (Haufe et al., 2014).

As a measure of LS contribution to the assignment to a particular class, we considered normalized L1-norm of the output weights for this class, corresponding to the given LS on the final classification layer of LF-CNN:

(4) $$w_{s}^{c}=\frac{\sum_{j=1}^{t}\left|w_{j,s}^{c}\right|}{\sum_{i=1}^{k}\sum_{j=1}^{t}\left|w_{j,i}^{c}\right|},$$

where *w* ∈ *ℝ*^*t*×*k*×2^ is output weights matrix, *c* = 0, 1 is a class index corresponding to spontaneous and voluntary class, respectively, and *s* is LS index.

**FIGURE 3:**
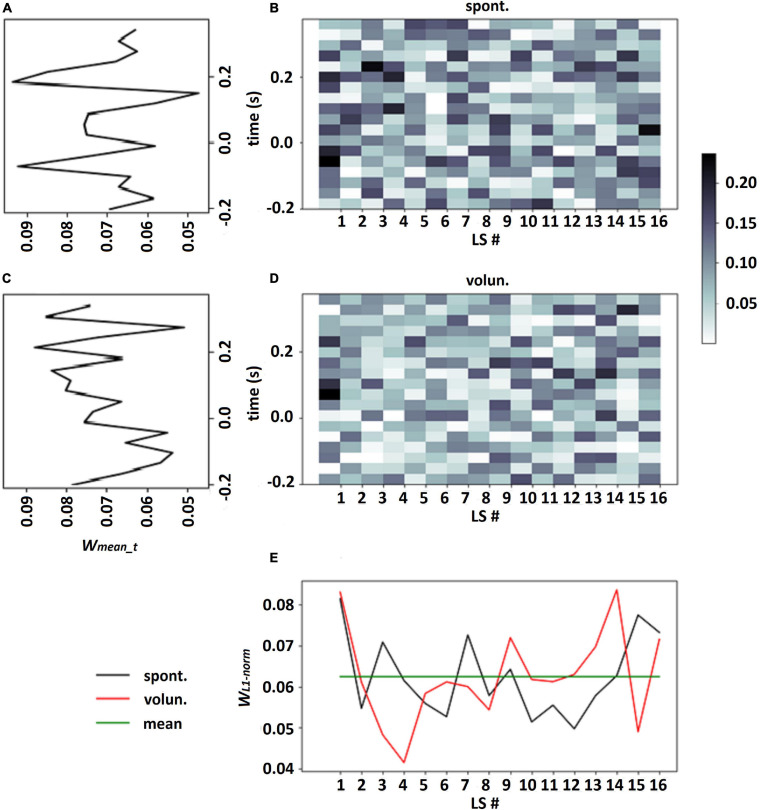
Output weights of LF-CNN learned on participant 114 data for spontaneous and voluntary classes (**B,D**, respectively), LS weights for each class according to eq. 4, where proportion of the L1-norm of output weights over time points in the total sum of L1-norms of all LSs **(E)**, averaged weights in every time point over all LSs for spontaneous and voluntary classes (**A,C**, respectively).

It should be emphasized that choosing a single LS which had the largest contribution to each class assignment may be incorrect especially in the case when all LSs have approximately equal contributions to classification: see an example in Figure 3E, where the calculation of the normalized L1 norm of the output weights for every LS revealed fluctuation in the vicinity of weight mean *w*_*mean*_ = 1/*k*. This observation confirmed our suggestion that all LSs should be taken into account in the analysis of LS patterns.

As the matrix W in eq. 2 transforms data from the signal space *X* ∈ *ℝ*^*n*×*t*^ into data from the source space (see Figure 2), it is reasonable to assume that obtained source data belongs to the subspace of useful signal for classification. To assess the amount of spatial information, utilized by LF-CNN, the fraction of explained variance (FVE) in every gradiometer was used as,

(5) $$F\,V\,E_{i}={\left(r\,\left(X_{i},X_{r\,e\,c_{i}}\right)\right)}^{2},i=1\,\ldots \,n,$$

$$X_{r\,e\,c}=A\,\hat{S,}$$

where *X*_*i*_,*X*_*rec*_*i*__ is input signal and reconstructed signal in *i*-th gradiometer, *r* is Pearson correlation coefficient.

### CNN Testing Algorithms

To assess the quality of the classification, nested cross-validation (CV) with five folds was utilized. There were two loops of CV, one inside another. The inner loop corresponded to 4-fold CV (dataset was divided into train/validation subset in ratio 3:1) and was used to determine the optimal number of the training iterations. It was defined as the average number of training iterations, where the maximum AUC value was achieved on a given fold, over all CV folds. The outer loop was 5-fold CV, used to assess the classifier performance on the test subset. Such organized CV with several times testing was preferable in order to obtain a more stable estimation of classifier performance as in the situation of small dataset neural networks tend to overfitting and classification results may vary when data are splitted anew.

Estimation of the CNN performance on the test dataset was carried out in two different ways, by a naïve approach and by means of a network ensemble.

(1) In the case of the naive method, after obtaining the optimal number of training iterations CNN was retrained anew, using the obtained iterations number, on the entire training sample. The trained network was then used to calculate ROC AUC on the test sample in each of the five folds of outer CV loop.

(2) To construct an ensemble of the neural networks, we used the weights obtained on each fold of inner CV loop. Thus, an ensemble of 4 CNNs was obtained. Their predictions on the test subset were averaged to calculate the target class probability. The obtained probabilities for each MEG trial from the test set were used for classification.

A permutation test was applied to examine whether the classifier had learned a significant predictive pattern in the data, that is, a real connection between the data and the class labels (Ojala and Garriga, 2010). The null hypothesis (*H*_0_) was that the features and the labels were independent (there was no difference between the classes). The distribution of the ROC AUC values under this null hypothesis was estimated by permuting the labels of the test data set. We used 1000 permutation for examining a single classification result. *P*-values were calculated for each of the five testing folds (Ojala and Garriga, 2010).

## Results

**FIGURE 4:**
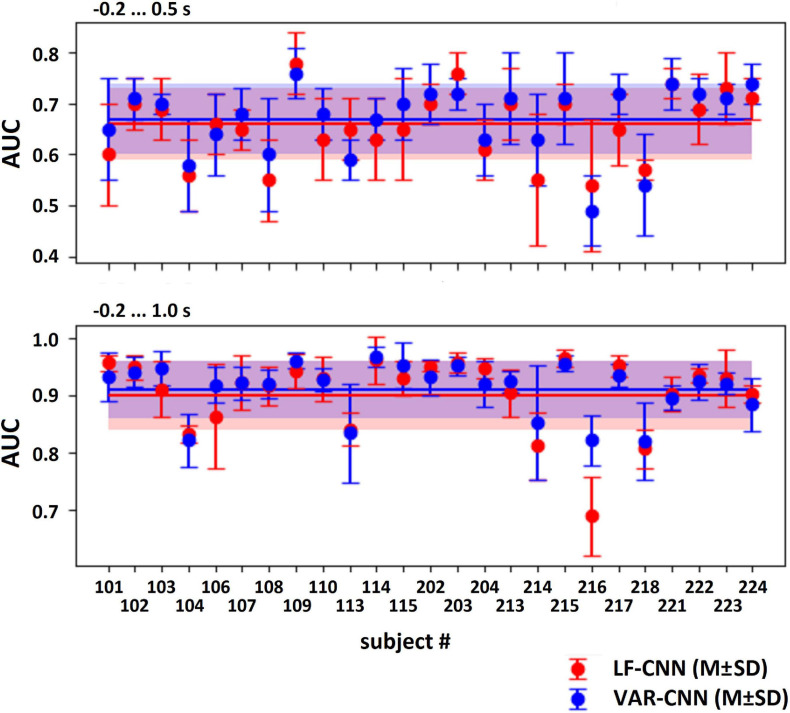
AUC values (*M* ± SD) for both CNNs, computed by naïve testing method for the test subsets of every participant.

The results of LF-CNN application to the MEG accompanying spontaneous and voluntary eye fixations in the gaze-controlled game are presented, per individual, in Table 1. Corresponding results of VAR-CNN are presented in Supplementary Table 1. CNNs performance, obtained by naïve testing method, is visualized on Figure 4 for both time intervals. Group statistics for both CNNs is given in Table 2.

**TABLE 1:** Individual classification performance of LF-CNN for 25 participants.

Subj. ID	Number of trials per class	Performance on original data			Result of permutation test
		AUC_*val*_	AUC_*na*__ï__*ve*_	AUC_*ensemble*_	
101	104	0.67 ± 0.03	0.60 ± 0.10	0.61 ± 0.07	
102	133	0.73 ± 0.04	0.70 ± 0.05	0.72 ± 0.06	
103	110	0.67 ± 0.03	0.69 ± 0.06	0.69 ± 0.05	
104	123	0.62 ± 0.03	0.56 ± 0.07	0.56 ± 0.07	
106	119	0.64 ± 0.04	0.66 ± 0.06	0.63 ± 0.07	
107	141	0.66 ± 0.02	0.65 ± 0.04	0.66 ± 0.04	
108	100	0.63 ± 0.03	0.55 ± 0.08	0.53 ± 0.05	–
109	95	0.73 ± 0.04	0.78 ± 0.06	0.76 ± 0.07	+
110	150	0.72 ± 0.01	0.63 ± 0.08	0.63 ± 0.04	
113	97	0.62 ± 0.07	0.65 ± 0.06	0.64 ± 0.04	
114	129	0.71 ± 0.03	0.63 ± 0.08	0.67 ± 0.08	
115	101	0.69 ± 0.04	0.65 ± 0.10	0.65 ± 0.10	
202	205	0.72 ± 0.03	0.70 ± 0.04	0.73 ± 0.02	+
203	217	0.74 ± 0.03	0.76 ± 0.04	0.75 ± 0.04	+
204	124	0.69 ± 0.02	0.61 ± 0.06	0.65 ± 0.03	
213	111	0.69 ± 0.03	0.70 ± 0.07	0.69 ± 0.06	
214	70	0.62 ± 0.06	0.55 ± 0.13	0.55 ± 0.14	–
215	177	0.71 ± 0.02	0.70 ± 0.04	0.73 ± 0.06	+
216	81	0.56 ± 0.02	0.54 ± 0.13	0.52 ± 0.15	–
217	157	0.67 ± 0.03	0.65 ± 0.07	0.68 ± 0.09	
218	76	0.60 ± 0.05	0.57 ± 0.02	0.54 ± 0.07	–
221	196	0.72 ± 0.03	0.74 ± 0.03	0.76 ± 0.04	+
222	165	0.73 ± 0.04	0.69 ± 0.07	0.69 ± 0.04	
223	98	0.69 ± 0.02	0.73 ± 0.07	0.72 ± 0.08	
224	137	0.75 ± 0.04	0.71 ± 0.04	0.72 ± 0.04	+
*M* ± SD	128 ± 38	0.67 ± 0.05	0.66 ± 0.07	0.66 ± 0.07	

*ROC AUCs computed for the test subsets on time interval –0.2 … 0.5 s using the naïve and ensemble testing procedures are denoted as AUC_*na*__ï__*ve*_ and AUC_*ensemble*_, respectively. AUC_*val*_ corresponds to mean AUC over cross-validation folds. All AUCs values are presented as *M* ± SD computed over cross-validation and test folds (for details see section “CNN Testing Algorithms”).*

*“+” denotes the participants with *p* < 0.05 on all testing folds (*H*_0_ hypothesis was always rejected, i.e., the difference from random performance was found for all folds) for both testing procedures.*

*“–” denotes the participants with *p* > 0.05 on all testing folds (*H*_0_ hypothesis was always accepted, i.e., the difference from random performance was not found for any fold) for both testing procedures.*

**TABLE 2:** Group mean ROC AUC values (*M* ± SD).

Time interval, s	LF-CNN		VAR-CNN	
	AUC_*na*__ï__*ve*_	AUC _*ensemble*_	AUC_*na*__ï__*ve*_	AUC _*ensemble*_
−0.2…0.5	0.66 ± 0.07	0.66 ± 0.07	0.67 ± 0.07	0.67 ± 0.07
−0.2…1.0	0.90 ± 0.06	0.90 ± 0.06	0.91 ± 0.05	0.91 ± 0.05

*AUCs computed for the test subsets using the naïve and ensemble testing procedures are denoted as AUC_*na*__ï__*ve*_ and AUC_*ensemble*_, respectively.*

The application of both CNNs resulted in a similar group mean AUC values. Individual AUC strongly varied across the group for both CNNs. LF-CNN and VAR-CNN demonstrated significantly nonrandom classification in all test folds for 6 and 8 participants, respectively. Only in a few participants (4 in the case of LF-CNN and 3 in the case of VAR-CNN) was nonrandom performance not achieved in any test fold. Notably, all these participants had low number of available fixations (not higher than 100). Pearson correlation coefficient showed significant positive linear correlation between the naïve/ensemble testing AUC and the number of trials per class both in the case of LF-CNN (*r* = 0.55/0.64, *p* = 0.0049/0.0005) and in the case of VAR-CNN (*r* = 0.57/0.60, *p* = 0.0030/0.0014), i.e., the more data were available for training, the better were the observed classification performance. For all participants strong overfitting of the CNNs were observed: mean group AUC values were about 0.98 on the training subset. ROC AUC curves for training and testing procedures are represented on Supplementary Figure 1 in Supplementary Data Sheet 1 for datasets of three subjects containing more than 100 trials per class: the first one (subject 104), revealed below group classification performance; the second one (subject 114) with AUC values near group average; and the third one (subject 221) with above mean AUC values.

Table 2, among other group statistics, includes AUCs also for the time interval extended beyond the time when the participants received the visual feedback to their gaze fixations. Extension of the interval led to a much better single-trial MEG classification (AUC > 0.9), confirming the good ability of both CNNs to discriminate the data. Wilcoxon matched pairs signed-ranks test comparing classification performance of LF-CNN and VAR-CNN for each type of testing procedure did not reveal a statistically significant difference between the results of CNN applications.

**FIGURE 5:**
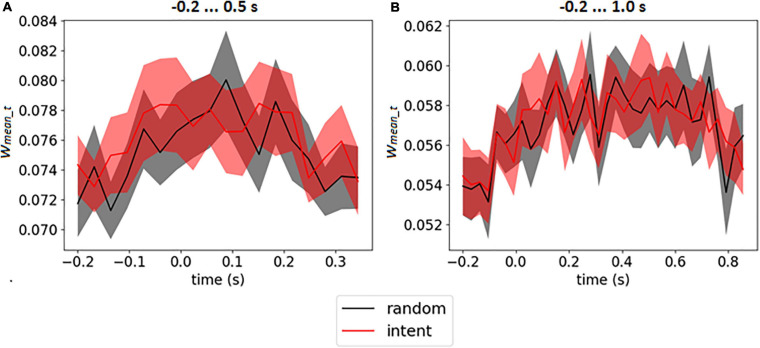
Mean output weights of LF-CNN in every time point averaged over participants for intervals –0.2 … 0.5 s **(A)** and 0.2 … 1.0 s **(B)**. The filled area corresponds to 95% confidence interval.

The weight matrices of the output layer (see an example in Figures 3B,D) and mean weights at every time point (see in Figures 3A,C) revealed no assignment of large weights to particular time points. It should be emphasized that for time interval −0.2 … 1.0 s (which included the feedback) the CNN did not assign larger weights for time points after 0.5 s, when feedback was received (see similar picture for enlarged interval on Supplementary Figure 2 in Supplementary Data Sheet 1). The mean weights of the time points over the group of the participants are presented on Figure 5 for both intervals.

We assessed that the full fraction of explained variance (FVE), computed for signal reconstructed from spatially filtered data as the share of the initial information passed through the spatial filter, was 35.12% and 31.92% in the case of intervals −0.2 … 0.5 s and 0.2 … 1.0 s, respectively, indicating that a significant part of the spatial information was utilized by the classifier.

To identify spatial features that contributed to the assignment of a given epoch to a particular class and to assess the possible contribution of the eye movement artifacts to classification results, the weights in matrix *A* (see eq. 3) of the LF-CNN’s LSs, weighted according to their contribution to each class, were visualized. We assumed that the topographical maps would present spatial patterns of features, most useful for the classifier, and that if they showed increased weights for frontal sensors, it would point out that the classifier heavily relied on eye movement artifacts, which mostly affect these sensors. Note that using gaze features to classify the voluntary and spontaneous gaze fixations could be, in principle, beneficial, but it deserves an additional study that should consider different issues, such as possible differences in gaze behavior patterns between the data used for classifier training and its application. At the current stage of the EBCI development it seemed reasonable to consider the classification of MEG data alone.

To take into account all LSs obtained we considered two variants of LSs averaging: weighted averaging, when weights were calculated by eq. 4 for each class separately, was proposed to reveal averaged patterns of each class, and mean pattern calculation, when every LS had equal weight *w*_*mean*_. Spatial patterns obtained by sets of averaging weights, corresponding to distinct classes, appeared to be almost equal which is consistent with the observation that the weights for each class determined by eq. 4 are close to *w*_*mean*_. While this result was observed for all participants, further in the paper we will illustrate only mean patterns over LSs, assuming that presented results were also obtained for the case of the weighted averaging. The mean pattern did not take into account LSs contributions to classification, therefore, the mean pattern could be used as an estimate of the common pattern of the subspace of the useful signal from LF-CNN’s point of view.

**FIGURE 6:**
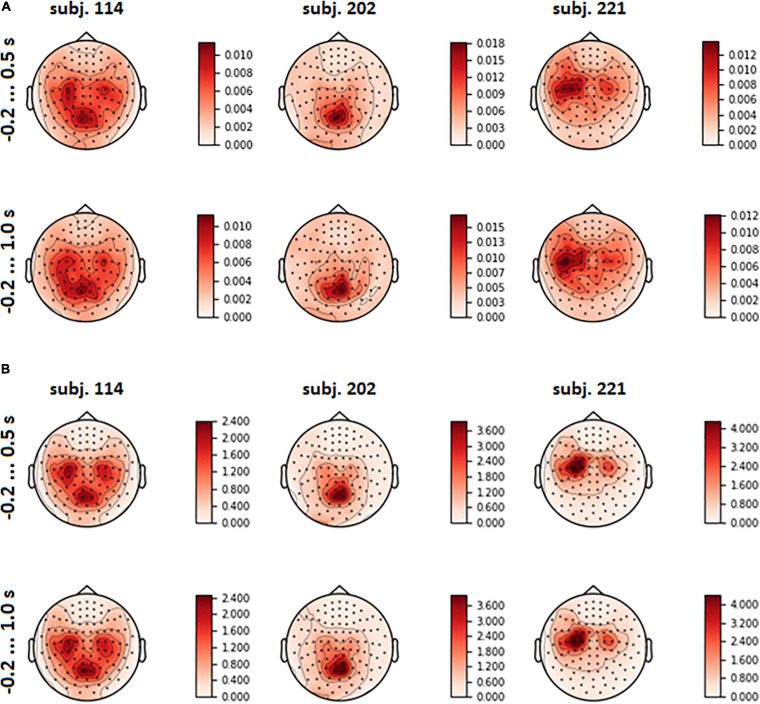
Mean spatial pattern over LSs, averaged over test folds, for three participants who revealed distinct types of the patterns **(A)**; input signal variance **(B)**.

**FIGURE 7:**
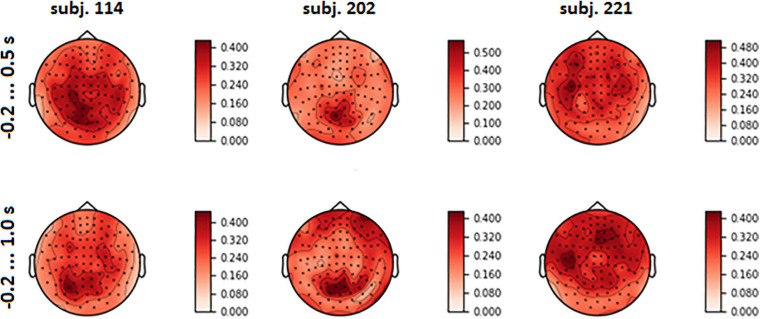
Topographical maps of full explained variance fraction (FVE) for the same participants as in Figure 6 (For the maps for other participants, see Supplementary Figure 3 in Supplementary Data Sheet 1).

The spatial patterns observed in the scalp projection maps (based on the location of gradiometers) of different participants revealed three dominant types: medial parietal areas, lateral central and pre-central areas of cortex (both left and right or mostly left) and joint pattern, including two first types (see Figure 6A). It was found out that patterns were strongly affected by the variance of the input signal (see Figure 6B and eq. 3). Variance influence caused the stability of spatial patterns obtained for the pre-feedback interval and for the interval with the interface feedback. To reduce variance impact, we calculated FVE in every gradiometer according to eq. 5. Examples of the FVE scalp topography patterns are presented in Figure 7.

**FIGURE 8:**
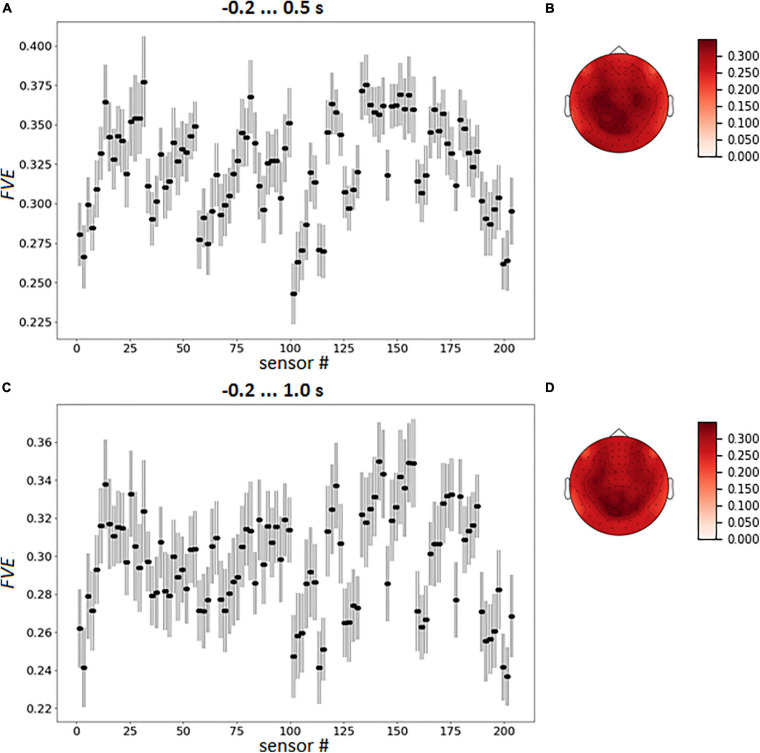
Group mean FVE patterns, as point plot with 95% confidence intervals for every gradiometer **(A,C)** and as topographical maps **(B,D)**. The data were averaged over test folds for each participant prior to other computations.

Visual analysis of FVE topographies (Figures 7, 8 and Supplementary Figure 3 in Supplementary Data Sheet 1) showed that areas with high FVE were localized primarily over left motor and pre-motor areas of the cortex, expanding in the rostral direction to the frontal and medial parietal lobe. This fact was consistent with the observations made in the statistical comparison of MEG amplitudes related to the same voluntary and spontaneous fixation data, where the most pronounced difference between them on the group level was also found in the left hemisphere. Note that spatial patterns did not change significantly with the enlargement of the time interval.

To capture the variation of sensor loadings the averaged pattern of FVE was calculated over the participants (Figure 8).

**FIGURE 9:**
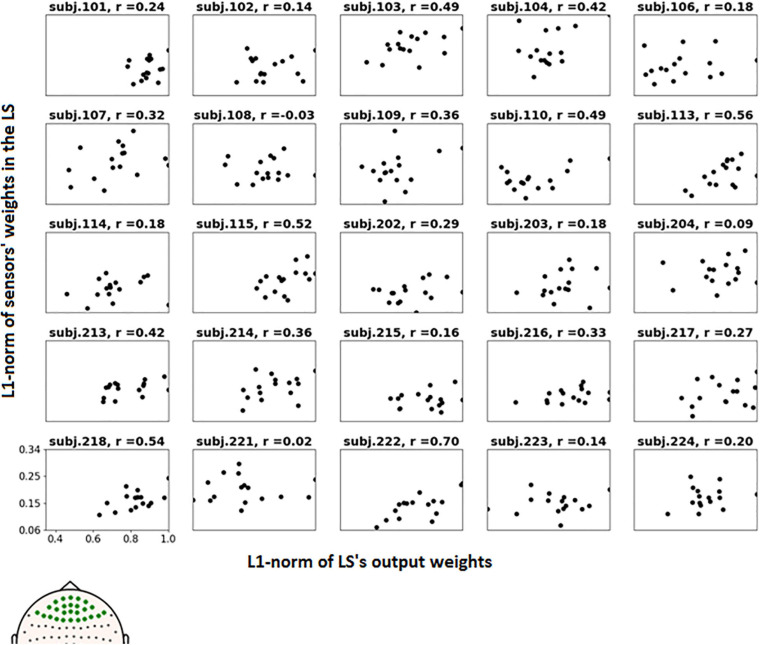
Dependence of the L1-norm of LS frontal sensor weights on L1-norm of LS output weights for the voluntary class. Each picture corresponds to a single participant, dots correspond to LSs. Positions of the frontal sensors (presumably most vulnerable to the eye movement), whose weights were averaged, are shown in bold on the head map in the left bottom corner.

**FIGURE 10:**
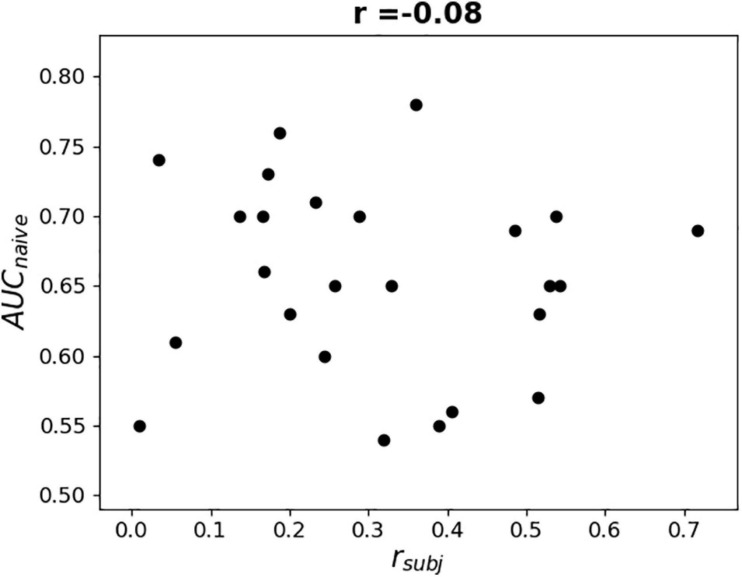
Dependence of AUC obtained by naïve testing procedure on Pearson correlation coefficient value between L1-norm of frontal sensors weights in each LS and L1-norm of LS’s output weights (see Figure 9).

To further examine the possible use of eye movement artifacts by the classifier, the dependence of L1-norm of frontal sensor weights in each LS on L1-norm of LS output weights, i.e., the contribution of given LS to classification, was plotted for all participants (Figure 9). We expected that if the artifacts made a significant contribution to the classification results, the plot should reveal some relationship between these variables, especially in participants with high classification performance, or at least outliers in the upper right corner of the plots (meaning that high weights related to the frontal MEG sensors corresponded to highest class separation ability). Although some patterns of this kind appeared and the corresponding Pearson correlation coefficient values were relatively high (see plots and correlation coefficients for the participants 110, 113, 115, 218, and 222 in Figure 9), group analysis revealed no relation between individual correlation coefficients and classifier performance (see Figure 10). Thus, although the contribution from the eye movement artifacts to the classification performance could not be ruled out in some participants, this contribution could be considered negligible at the group level.

## Discussion

In this study, MEG co-registered with the eye tracker data was utilized to distinguish voluntary eye fixations used for sending commands to a computer from spontaneous ones. Above-chance classification results were obtained for 21 out of 25 participants using 700 ms (−0.2 … 0.5 s) epochs of MEG data centered on the onset of the fixation. AUC values (*M* ± SD) for naïve and ensemble testing procedures appeared to be the same and equal to 0.66 ± 0.07 and 0.67 ± 0.07 for LF-CNN and VAR-CNN, respectively.

Extending MEG epochs up to 1000 ms from the fixation onset to include the evoked response to the visual feedback to fixation improved the classification performance dramatically (Table 2) for both CNN architectures: for LF-CNN AUC appeared to be 0.91 ± 0.06 and 0.91 ± 0.06, for VAR-CNN – 0.91 ± 0.04 and 0.91 ± 0.05 for naïve and ensemble testing, respectively.

Spatial patterns informative for classification with LF-CNN were localized mainly over left motor and pre-motor areas expanding in the rostral direction in the frontal lobe and over the medial parietal lobe, suggesting that in both cases (expectation and processing of the visual feedback to voluntary saccade) classification was primarily based on the activity from brain areas controlling eye movement, such as the frontal and parietal eye fields (FEF, PEF) (Schall and Thompson, 1999; Medendorp et al., 2011). It is tempting to propose, based on such good single-trial performance, a new EBCI that could utilize the data from this enlarged time interval. However, gaze behavior in this interval alone might be already enough to reliably distinguish voluntary and spontaneous fixations, and it is indeed utilized in some of the existing gaze-based input systems. Moreover, the use of this interval would imply that a response to feedback should be required in the online implementation of the EBCI, which clearly contradicts our goal to create an interface for fluent human-machine interaction, in which the action should be triggered by gaze directly, without the need to make any additional confirmation actions.

Interestingly, informative spatial patterns (Haufe et al., 2014) obtained from the model weights learned from shorter (−0.2 … 0.5 s) and longer (−0.2 … 1 s) segments of the MEG data generally remained the same within participants (Figure 6A). One explanation for this could be that in both cases activation pattern estimates were strongly affected by the covariance structure of the data. Because sensors with greater variance have higher SNR, it is reasonable to assume that these same sensors were also the most informative for classification. At the same time, because the inhomogeneity of the spatial variance may have affected our estimates of the contribution of the oculomotor artifacts to the performance of the classifier, we recommend caution when interpreting these results.

To reduce variance impact, we assessed the fraction of variance extracted by the spatial filter from each gradiometer. The resulting FVE spatial patterns differed slightly for cases of the pre-feedback and enlarged time intervals (Figures 7, 8). It is still difficult to fully exclude the usage of eye movements by the CNNs, but analysis of spatial filters contributing to LF-CNN performance revealed no signs of substantial eye movement contribution. Spatial patterns of weights and FVE did not reveal maxima in frontal sensors and, therefore, provided no evidence for the use of eye movement artifacts by the classifier. At the same time, a full FVE obtained for signal reconstructed from spatially filtered data showed that 35.12% and 31.92% of the input information passed through the spatial filter and was utilized by the classifier in case of intervals −0.2 … 0.5 s and −0.2 … 1.0 s, respectively. Thus, the LF-CNN network likely classified the voluntary and spontaneous eye fixations based mainly on MEG components originating from brain sources and not oculomotor activity.

As we made no prior assumptions regarding the MEG signatures that might inform the classification, we were interested in spatiotemporal features of the signal that were extracted from MEG by CNN. It should be also emphasized, that the pattern obtained from the forward model does not reflect the full spatiotemporal data/feature structure, but only shows from where the signal features were taken by the classifier. One way to assess the involvement of signal features in classification is to estimate the amount of variance (FVE in the article) shared by the original signal and the source subspace extracted by CNN. The CNN’s source subspace can differ significantly from actual signal sources in the brain which are differently activated in the two classes. It should be considered as subspace of features, useful for a given classifier (Weichwald et al., 2015). FVE metric allows us to estimate the fraction of information, utilized by classifiers from every gradiometer. Thus, obtained patterns should be considered as maps of features selected by the classifier. In an earlier study, where the EEG was co-registered with eye tracker data in a similar paradigm, ROC AUC was 0.69 ± 0.09 (Shishkin et al., 2016), i.e., close to the results obtained in this study, and these results were improved with the use of a CNN (Kozyrskiy et al., 2018). Note that perfect single-trial classification is not necessary: as proposed by Protzak et al. (2013), the classifier can be tuned to have high specificity and relatively low sensitivity, and in the case of a miss the user can just dwell at the same target for a longer time, so that the interface would detect the intention to click by applying the second dwell time threshold, this time without using the BCI part of the EBCI.

As it was already found in an online EEG-based study (Nuzhdin et al., 2017), this level of classifier performance is insufficient for online EBCI studies. There are, however, at least several ways in which classification can be improved upon:

(1) Joint use of MEG, EEG, and gaze data, for example, by fusing the outputs of the classifiers each of which works with the specific type of data. Gaze data are typically considered to be not different between voluntary gaze-based control and spontaneous gaze behaviors.
(2) Use of MEG and EEG spectral features. Some advanced methods of deep learning sensitivity analysis, e.g., the gradient-based attribution methods (Ancona et al., 2018) and methods for frequency spectral analysis in deep architectures developed for brain signals application in works (Schirrmeister et al., 2017; Hartmann et al., 2018) can also help to reveal discriminative signal features in the experiment paradigm described in this work.
(3) Use of more data for classifiers training, especially by using several sessions recorded on different days in the same participant or a large group of participants. The latter approach, i.e., transfer learning, was shown to be feasible for the CNNs used in this work (Zubarev et al., 2019). It did not provide improvement when applied to the data used in the current study (mean AUC value for leave-one-subject-out testing was 0.59; in case of training and testing on the joint dataset of all participants AUC was equal to 0.63), possibly due to higher variability of the MEG patterns or lower signal-to-noise ratio, but it is not unlikely that the classifier performance will improve with more participants involved.
(4) Changing the preprocessing pipeline for better handling of the single-trial data. In the current study, MEG raw data were preprocessed using the *MaxFilter* software that applied the tSSS procedure. As it is applied blockwise, this procedure could likely introduce certain irregularities to the signal that do not affect the averaged signal (typically used in the studies of phase-locked MEG components) but may make more difficult classification of the single-trial data. In online experiments, tSSS cannot be used due to the blockwise organization of processing and relatively large size of blocks needed for its effective work (typically of the order of seconds), thus a certain change of preprocessing is inevitable.

These approaches to improving classification (or at least those of them that will prove to be effective) can be combined, so that significant improvement of the classification performance seems likely.

## Conclusion

In this study, we attempted to determine if short single-trial segments of MEG data related to voluntary and spontaneous eye fixations can help to distinguish between such types of fixations. The adaptive CNNs, LF-CNN and VAR-CNN, developed recently for MEG data classification (Zubarev et al., 2019), were applied for binary classification of the MEG signals corresponding to spontaneous and voluntary eye fixations collected in participants who used voluntary fixations with 500 ms dwell time threshold to play a game. Nonrandom classification results were obtained with both CNNs for the majority of 25 participants using short (700 ms) MEG intervals without the formation of feature vectors (group *M* ± SD were 0.66 ± 0.07 for LF-CNN and 0.67 ± 0.07 for VAR-CNN). Analysis of spatial patterns contributing to classification did not reveal signs of significant eye movement contribution to the classification results. We conclude that the classification of MEG signals may help to determine the correctness of the responses of gaze-based interfaces to eye fixations on a single-trial basis. Current results of intention detection prior to gaze-based interface’s feedback are not sufficient for online single-trial eye fixation classification using MEG data alone, and further work is needed to find out if it could be used in practical applications.

## Data Availability Statement

The data analyzed in this study is subject to the following licenses/restrictions: We used the MEG data from another study by our group (Vasilyev et al., in prep.) recorded at the MEG Center, Moscow State University for Psychology and Education. These data are available on a reasonable request. Requests to access these datasets should be directed to corresponding author (SS).

## Ethics Statement

The studies involving human participants were reviewed and approved by the Ethical Committee of the Moscow State University of Psychology and Education. The patients/participants provided their written informed consent to participate in this study.

## Author Contributions

AO, AV, IZ, BK, and SS were responsible for the concept. AO performed the data analysis and wrote the manuscript. AV analyzed the eye gaze data. AV, IZ, BK, and SS revised the manuscript. All authors contributed to the article and approved the submitted version.

## Conflict of Interest

The authors declare that the research was conducted in the absence of any commercial or financial relationships that could be construed as a potential conflict of interest.

## Footnotes

1 https://github.com/hyperopt/hyperopt

## References

[B1] Allison B. Graimann B. Gräser A. (2007). “Why use a BCI if you are healthy,” in *BRAINPLAY 07 Brain-Computer Interfaces and Games Workshop at ACE (Advances in Computer Entertainment) 2007*, (German: IAT), 7–11.

[B2] Ancona M. Ceolini E. Öztireli C. Gross M. (2018). *Towards Better Understanding of Gradient-Based Attribution Methods for Deep Neural Networks. Arxiv [preprint].* Available online at: https://arxiv.org/abs/1711.06104 (accessed on November 29, 2020).

[B3] Blankertz B. Acqualagna L. Dähne S. Haufe S. Schultze-Kraft M. Sturm I. (2016). The Berlin brain-computer interface: progress beyond communication and control. *Front. Neurosci.* 10:530. 10.3389/fnins.2016.00530 27917107 PMC5116473

[B4] Borna A. Carter T. R. Colombo A. P. Jau Y. Y. McKay J. Weisend M. (2020). Non-invasive functional-brain-imaging with an OPM-based magnetoencephalography system. *PLoS One* 15:e0227684. 10.1371/journal.pone.0227684 31978102 PMC6980641

[B5] Borna A. Carter T. R. Goldberg J. D. Colombo A. P. Jau Y.-Y. Berry C. (2017). A 20-channel magnetoencephalography system based on optically pumped magnetometers. *Phys. Med. Biol.* 62:8909. 10.1088/1361-6560/aa93d1 29035875 PMC5890515

[B6] Boto E. Bowtell R. Krüger P. Fromhold T. M. Morris P. G. Meyer S. S. (2016). On the Potential of a New Generation of Magnetometers for MEG: A Beamformer Simulation Study. *PLoS One* 11:e0157655. 10.1371/journal.pone.0157655 27564416 PMC5001648

[B7] Boto E. Hill R. M. Rea M. Holmes N. Seedat Z. A. Leggett J. (2020). *Measuring Functional Connectivity with Wearable Meg. Biorxiv. [preprint].* Available online at: https://www.biorxiv.org/content/10.1101/2020.09.25.313502v1 (accessed on November 29, 2020). 10.1016/j.neuroimage.2021.117815 PMC8216250 33524584

[B8] Boto E. Holme N. Leggett J. Roberts G. Shah V. Meyer S. S. (2018). Moving magnetoencephalography towards real-world applications with a wearable system. *Nature* 555 657–661. 10.1038/nature26147 29562238 PMC6063354

[B9] Boto E. Meyer S. S. Shah V. Alem O. Knappe S. Kruger P. (2017). A new generation of magnetoencephalography: Room temperature measurements using optically-pumped magnetometers. *NeuroImage* 149:414. 10.1016/j.neuroimage.2017.01.034 28131890 PMC5562927

[B10] Boto E. Seedat Z. A. Holmes N. Leggett J. Hill R. M. Roberts G. (2019). Wearable neuroimaging: Combining and contrasting magnetoencephalography and electroencephalography. *NeuroImage* 201:116099. 10.1016/j.neuroimage.2019.116099 31419612 PMC8235152

[B11] Cinel C. Valeriani D. Poli R. (2019). Neurotechnologies for human cognitive augmentation: current state of the art and future prospects. *Front. Hum. Neurosci.* 13:13. 10.3389/fnhum.2019.00013 30766483 PMC6365771

[B12] Color Lines (2003). *Color Lines for DOS (1992) - MobyGames.* Available online at: https://www.mobygames.com/game/dos/color-lines (accessed on December 02, 2020).

[B13] Ettinger U. Ffytche D. H. Kumari V. Kathmann N. Reuter B. Zelaya F. (2008). Decomposing the neural correlates of antisaccade eye movements using event-related fMRI. *Cerebral Cortex* 18:1159. 10.1093/cercor/bhm147 17728263

[B14] Hallett P. E. (1978). Primary and secondary saccades to goals defined by instructions. *Vis. Res.* 10:1296 10.1016/0042-6989(78)90218-3 726270

[B15] Hartmann K. G. Schirrmeister R. T. Ball T. (2018). “Hierarchical internal representation of spectral features in deep convolutional networks trained for EEG decoding,” in *6th Int. Conf. on Brain–Computer Interface (IEEE)*, (New York: IEEE), 1–6.

[B16] Haufe S. Meinecke F. Görgen K. Dähne S. Haynes J. D. Blankertz B. (2014). On the interpretation of weight vectors of linear models in multivariate neuroimaging. *NeuroImage* 87:110. 10.1016/j.neuroimage.2013.10.067 24239590

[B17] Hill R. M. Boto E. Holmes N. Hartley C. Seedat Z. A. Leggett J. (2019). A tool for functional brain imaging with lifespan compliance. *Nat. Commun*. 10:4785. 10.1038/s41467-019-12486-x 31690797 PMC6831615

[B18] Hill R. M. Boto E. Rea M. Holmes N. Leggett J. Coles L. A. (2020). Multi-channel whole-head OPM-MEG: Helmet design and a comparison with a conventional system. *Neuroimage* 219:116995. 10.1016/j.neuroimage.2020.116995 32480036 PMC8274815

[B19] Ihme K. Zander T. O. (2011). “What you expect is what you get? Potential use of contingent negative variation for passive BCI systems in gaze-based HCI,” in *International Conference on Affective Computing and Intelligent Interaction*, (Berlin: Springer), 447–456. 10.1007/978-3-642-24571-8_57

[B20] Iivanainen J. Stenroos M. Parkkonen L. (2017). Measuring MEG closer to the brain: Performance of on-scalp sensor arrays. *NeuroImage* 147:553. 10.1016/j.neuroimage.2016.12.048 28007515 PMC5432137

[B21] Iivanainen J. Zetter R. Grön M. Hakkarainen K. Parkkonen L. (2019). On-scalp MEG system utilizing an actively shielded array of optically-pumped magnetometers. *Neuroimage* 194:258. 10.1016/j.neuroimage.2019.03.022 30885786 PMC6536327

[B22] Jacob R. J. K. (1990). What you look at is what you get: eye movement-based interaction techniques. *Proc. SIGCHI Conf. Hum. Fact. Comp. Sys.* 11:18 10.1145/97243.97246

[B23] Kozyrskiy B. L. Ovchinnikova A. O. Moskalenko A. D. Velichkovsky B. M. Shishkin S. L. (2018). Classification of the gaze fixations in the eye-brain-computer interface paradigm with a compact convolutional neural network. *Proc. Comput. Sci.* 145:299 10.1016/j.procs.2018.11.062

[B24] Lawhern V. J. Solon A. J. Waytowich N. R. Gordon S. M. Hung C. P. Lance B. J. (2016). *EEGNet: A Compact Convolutional Network for EEG-Based Brain-Computer Interfaces. arXiv [Preprint].* Available online at: https://arxiv.org/abs/1611.08024 (accessed on November 29, 2020). 10.1088/1741-2552/aace8c 29932424

[B25] Lawhern V. J. Solon A. J. Waytowich N. R. Gordon S. M. Hung C. P. Lance B. J. (2018). EEGNet: a compact convolutional neural network for EEG-based brain–computer interfaces. *J. Neural. Eng*. 15:056013. 10.1088/1741-2552/aace8c 29932424

[B26] Limes M. E. Foley E. L. Kornack T. W. Caliga S. McBride S. Braun A. (2020). *Total-Field Atomic Gradiometer for Unshielded Portable Magnetoencephalography. arXiv [Preprint]*. Available online at: https://arxiv.org/abs/2001.03534v1 (accessed on November 29, 2020).

[B27] Lotte F. Bougrain L. Cichocki A. Clerc M. Congedo M. Rakotomamonjy A. (2018). A review of classification algorithms for EEG-based brain–computer interfaces: a 10 year update. *J. Neural. Engin*. 15:031005. 10.1088/1741-2552/aab2f2 29488902

[B28] Luo W. Li Y. Urtasun R. Zemel R. (2017). *Understanding the Effective Receptive Field in Deep Convolutional Neural Networks. arXiv [Preprint]*. Available online at: https://arxiv.org/abs/1701.04128 (accessed on November 29, 2020).

[B29] Martins N. R. B. Angelica A. Chakravarthy K. Svidinenko Y. Boehm F. J. Opris I. (2019). Human Brain/Cloud Interface. *Front. Neurosci.* 13:112. 10.3389/fnins.2019.00112 30983948 PMC6450227

[B30] Medendorp W. P. Buchholz V. N. Van Der Werf J. Leoné F. T. M. (2011). Parietofrontal circuits in goal-oriented behaviour. *Eur. J. Neurosci.* 11:2027. 10.1111/j.1460-9568.2011.07701.x 21645097

[B31] Nijholt A. Van Erp J. B. Heylen D. K. (2008). BrainGain: BCI for HCI and games. 2008 AISB Symp. *Brain Comput. Interf. Hum. Comput. Interact.* 32:35.

[B32] Nuzhdin Y. O. Shishkin S. L. Fedorova A. A. Kozyrskiy B. L. Medyntsev A. A. Svirin E. P. (2017). Passive detection of feedback expectation: Towards fluent hybrid eye-brain-computer interfaces. *Graz. BCI Conf.* 361:366. 10.3217/978-3-85125-533-1-66 32992130

[B33] Ojala M. Garriga G. C. (2010). Permutation tests for studying classifier performance. *J. Mach. Learn. Res.* 11:1863 10.1109/icdm.2009.108

[B34] Paek A. Y. Kilicarslan A. Korenko B. Gerginov V. Knappe S. Contreras-Vidal J. L. (2020). *Towards a Portable Magnetoencephalography Based Brain Computer Interface with Optically-Pumped Magnetometers.* Berlin: Springer, 3420 3423 10.1109/EMBC44109.2020.9176159 33018738

[B35] Protzak J. Ihme K. Zander T. O. (2013). A passive brain-computer interface for supporting gaze-based human-machine interaction. *Proc. Int. Conf. UAHCI.* 662:671 10.1007/978-3-642-39188-0_71

[B36] Schall J. D. Thompson K. G. (1999). Neural selection and control of visually guided eye movements. *Annu. Rev. Neurosci*. 22:259. 10.1146/annurev.neuro.22.1.241 10202539

[B37] Schirrmeister R. T. Springenberg J. T. Fiederer L. D. J. Glasstetter M. Eggensperger K. Tangermann M. (2017). Deep learning with convolutional neural networks for EEG decoding and visualization. *Hum. Brain Mapp.* 11:5420. 10.1002/hbm.23730 28782865 PMC5655781

[B38] Shishkin S. L. Nuzhdin Y. O. Svirin E. P. Trofimov A. G. Fedorova A. A. Kozyrskiy B. L. (2016). EEG negativity in fixations used for gaze-based control: Toward converting intentions into actions with an Eye-Brain-Computer Interface. *Front. Neurosci.* 10:528. 10.3389/fnins.2016.00528 27917105 PMC5114310

[B39] Srivastava N. Hinton G. Krizhevsky A. Sutskever I. Salakhutdinov R. (2014). Dropout: a simple way to prevent neural networks from overfitting. *J. Mach. Learn. Res.* 15:1958. 10.5555/2627435.2670313 26215079

[B40] Taulu S. Simola J. (2006). Spatiotemporal signal space separation method for rejecting nearby interference in MEG measurements. *Phys. Med. Biol.* 51 1759–1768. 10.1088/0031-9155/51/7/008 16552102

[B41] The Ohba Analysis Group (2010). *MaxFilter User’s Guide.* Helsinki: Elekta Oy.

[B42] Weichwald S. Meyer T. Özdenizci O. Schölkopf B. Ball T. Grosse-Wentrup M. (2015). Causal interpretation rules for encoding and decoding models in neuroimaging. *NeuroImage* 110 48–59. 10.1016/j.neuroimage.2015.01.036 25623501

[B43] Zander T. O. Kothe C. (2011). Towards passive brain–computer interfaces: applying brain–computer interface technology to human–machine systems in general. *J. Neural. Eng.* 8:025005 10.1088/1741-2560/8/2/025005 21436512

[B44] Zhang R. Xiao W. Ding Y. Feng Y. Peng X. Shen L. (2020). Recording brain activities in unshielded Earth’s field with optically pumped atomic magnetometers. *Sci. Adv*. 6:eaba8792. 10.1126/sciadv.aba8792 32582858 PMC7292643

[B45] Zhang X. Chen C. Q. Zhang M. K. Ma C. Y. Zhang Y. Wang H. (2020). Detection and analysis of MEG signals in occipital region with double-channel OPM sensors. *J. Neurosci. Methods* 348:108948. 10.1016/j.jneumeth.2020.108948 32950554

[B46] Zubarev I. Zetter R. Halme H. L. Parkkonen L. (2019). Adaptive neural network classifier for decoding MEG signals. *Neuroimage* 425:434. 10.1016/j.neuroimage.2019.04.068 31059799 PMC6609925

