# Supplementary material for: MEG-Based Detection of Voluntary Eye Fixations Used to Control a Computer
Source: Front Neurosci. 2021 Feb 5;15:619591. doi: 10.3389/fnins.2021.619591 (PMC7892913; doi:10.3389/fnins.2021.619591)
Supplement: Supplementary file 1 [file Table_1.PDF]

Supplementary Table 1. Individual classification performance of VAR-CNN for 25 participants. AUCs computed for the test subsets on time interval –0.2 … 0.5 s using the naïve and ensemble testing procedures are denoted as $AUC_{naïve}$ and $AUC_{ensemble}$, respectively. AUC_{val} corresponds to mean AUC over cross-validation folds. All AUCs values are presented as M ± SD computed over cross-validation and tests folds (for details see *CNN testing algorithms* in the main text). "+" denotes the participants with $p < 0.05$ on all testing folds (H_0 hypothesis was always rejected, i.e., the difference from random performance was found for all folds) for both testing procedures. "–" denotes the participants with $p > 0.05$ on all testing folds (H_0 hypothesis was always accepted, i.e., the difference from random performance was not found for any fold) for both testing procedures.

Subj. ID	Number of trials per class	Performance on original data			Result of permutation test
		AUCval	AUCnaïve	AUC ensemble	
101	104	0.69±0.03	0.65±0.10	0.62±0.09	
102	133	0.75±0.02	0.71±0.04	0.70±0.03	+
103	110	0.70±0.04	0.70±0.02	0.70±0.05	
104	123	0.64±0.02	0.58±0.09	0.57±0.10	
106	119	0.63±0.02	0.64±0.08	0.62±0.11	
107	141	0.69±0.04	0.68±0.05	0.70±0.04	+
108	100	0.66±0.06	0.60±0.11	0.59±0.12	
109	95	0.78±0.04	0.76±0.05	0.75±0.06	+
110	150	0.72±0.03	0.68±0.05	0.68±0.06	
113	97	0.68±0.06	0.59±0.04	0.58±0.07	–
114	129	0.69±0.04	0.67±0.04	0.68±0.06	
115	101	0.67±0.05	0.70±0.07	0.69±0.09	
202	205	0.76±0.02	0.72±0.06	0.73±0.04	+
203	217	0.75±0.02	0.72±0.03	0.72±0.04	+
204	124	0.68±0.04	0.63±0.07	0.67±0.06	
213	111	0.71±0.03	0.71±0.09	0.71±0.09	
214	70	0.68±0.02	0.63±0.09	0.63±0.08	
215	177	0.73±0.02	0.71±0.09	0.72±0.11	
216	81	0.58±0.04	0.49±0.07	0.49±0.07	–
217	157	0.68±0.02	0.72±0.04	0.67±0.05	
218	76	0.62±0.03	0.54±0.10	0.54±0.12	–
221	196	0.73±0.02	0.74±0.05	0.74±0.05	+
222	165	0.75±0.03	0.72±0.03	0.71±0.02	+
223	98	0.75±0.02	0.71±0.03	0.68±0.02	
224	137	0.71±0.04	0.74±0.04	0.74±0.03	+
M ± SD	128±38	0.70±0.05	0.67±0.07	0.67±0.07	
