# Supplementary material for: MEG-Based Detection of Voluntary Eye Fixations Used to Control a Computer
Source: Front Neurosci. 2021 Feb 5;15:619591. doi: 10.3389/fnins.2021.619591 (PMC7892913; doi:10.3389/fnins.2021.619591)
Supplement: Supplementary file 2 [file Data_Sheet_1.PDF]

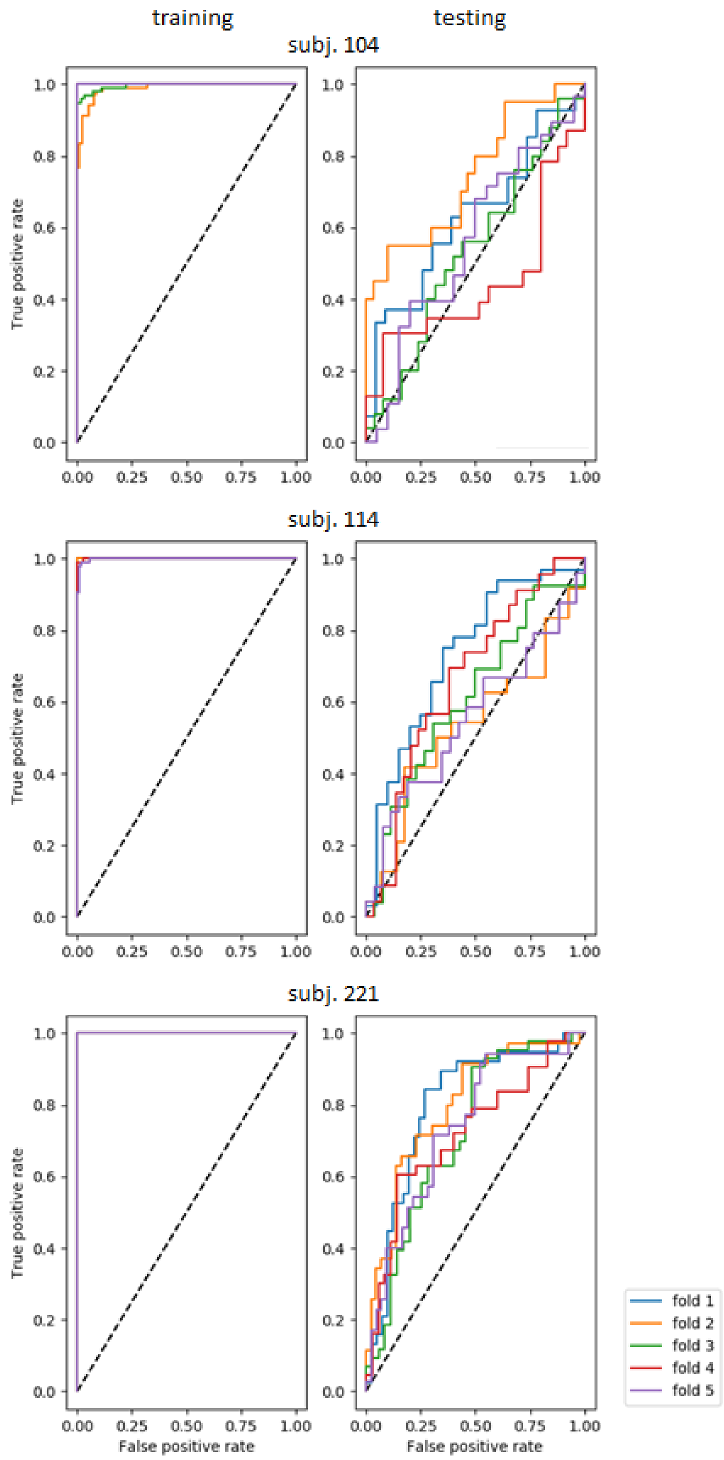

Supplementary Figure 1. ROC AUC curves for each fold of training (left) and testing (right) procedures, obtained on datasets of three subjects. Some lines may not be visible if they coincide with lines drawn on top of them.

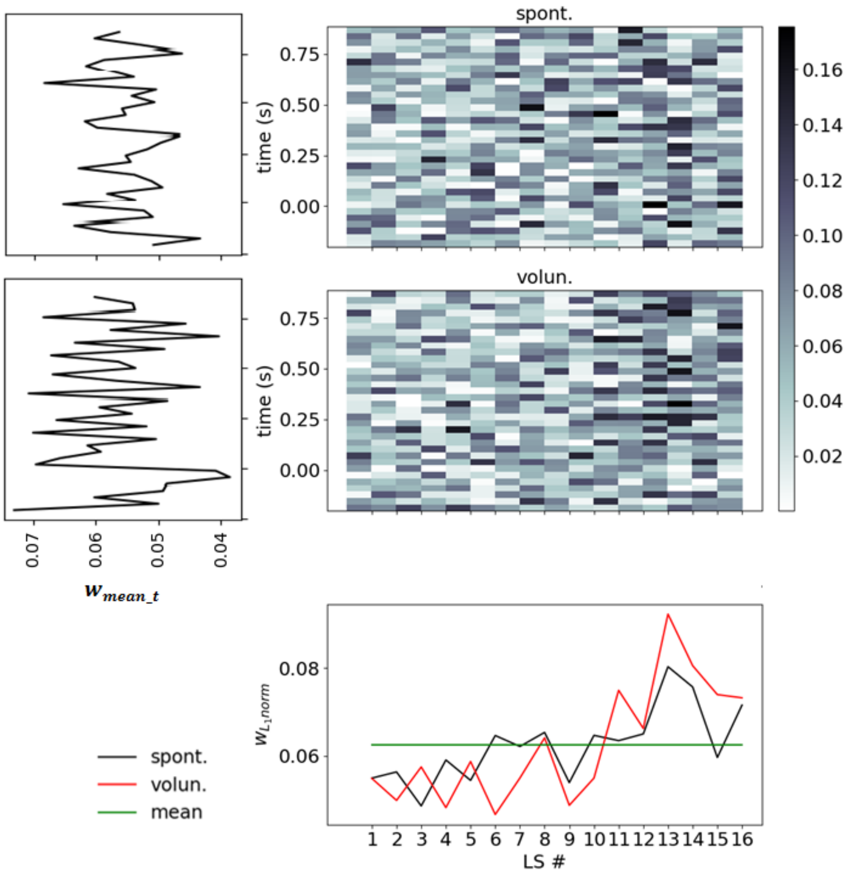

Supplementary Figure 2. Output weights of LF-CNN learned on participant 114 data in time interval -0.2 … 1.0 s for spontaneous and voluntary classes (top and middle, respectively), LS' weights for each class according to Equation 4, where proportion of the L1-norm of output weights over time points in total sum of L1-norms of all LSs (bottom), averaged weights in every time point over all LSs (left).

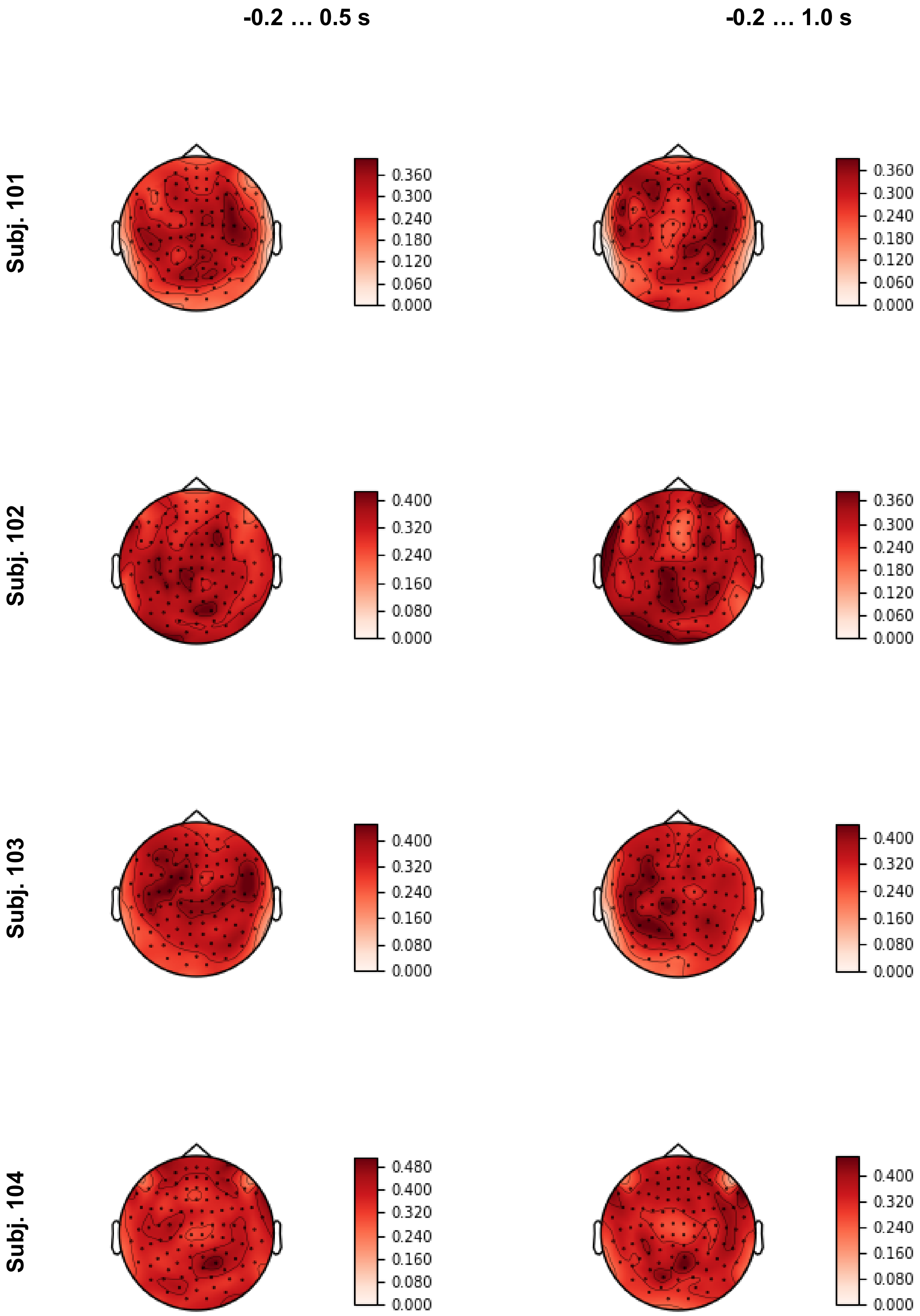
-0.2 … 0.5 s
-0.2 … 1.0 s
Subj. 101
0.360 0.300 0.240 0.180 0.120 0.060 0.000
0.360 0.300 0.240 0.180 0.120 0.060 0.000
Subj. 102
0.400 0.320 0.240 0.160 0.080 0.000
0.360 0.300 0.240 0.180 0.120 0.060 0.000
Subj. 103
0.400 0.320 0.240 0.160 0.080 0.000
0.400 0.320 0.240 0.160 0.080 0.000
Subj. 104
0.480 0.400 0.320 0.240 0.160 0.080 0.000
0.400 0.320 0.240 0.160 0.080 0.000

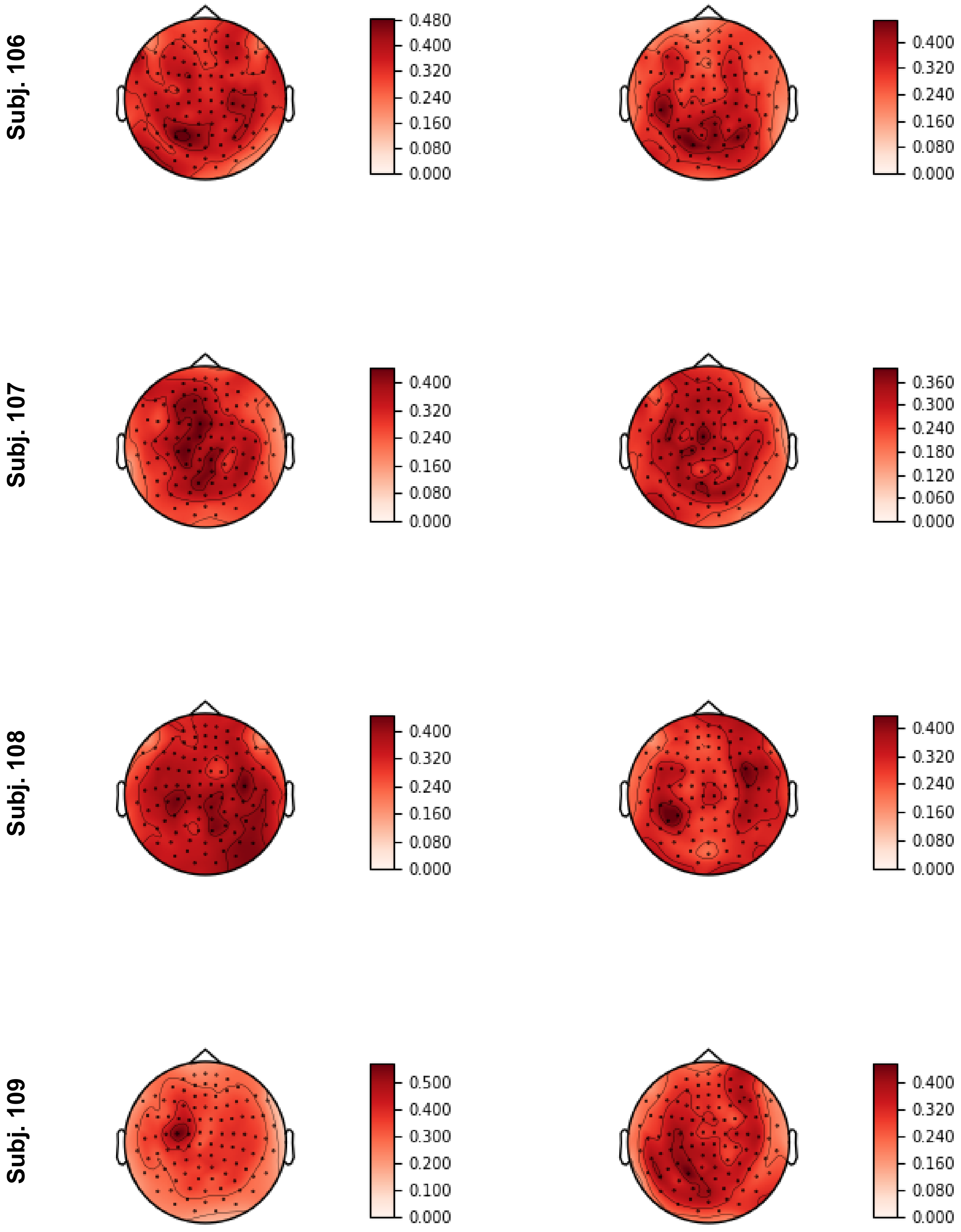

Subj. 106
0.480
0.400
0.320
0.240
0.160
0.080
0.000
0.400
0.320
0.240
0.160
0.080
0.000
Subj. 107
0.400
0.320
0.240
0.160
0.080
0.000
0.360
0.300
0.240
0.180
0.120
0.060
0.000
Subj. 108
0.400
0.320
0.240
0.160
0.080
0.000
0.400
0.320
0.240
0.160
0.080
0.000
Subj. 109
0.500
0.400
0.300
0.200
0.100
0.000
0.400
0.320
0.240
0.160
0.080
0.000

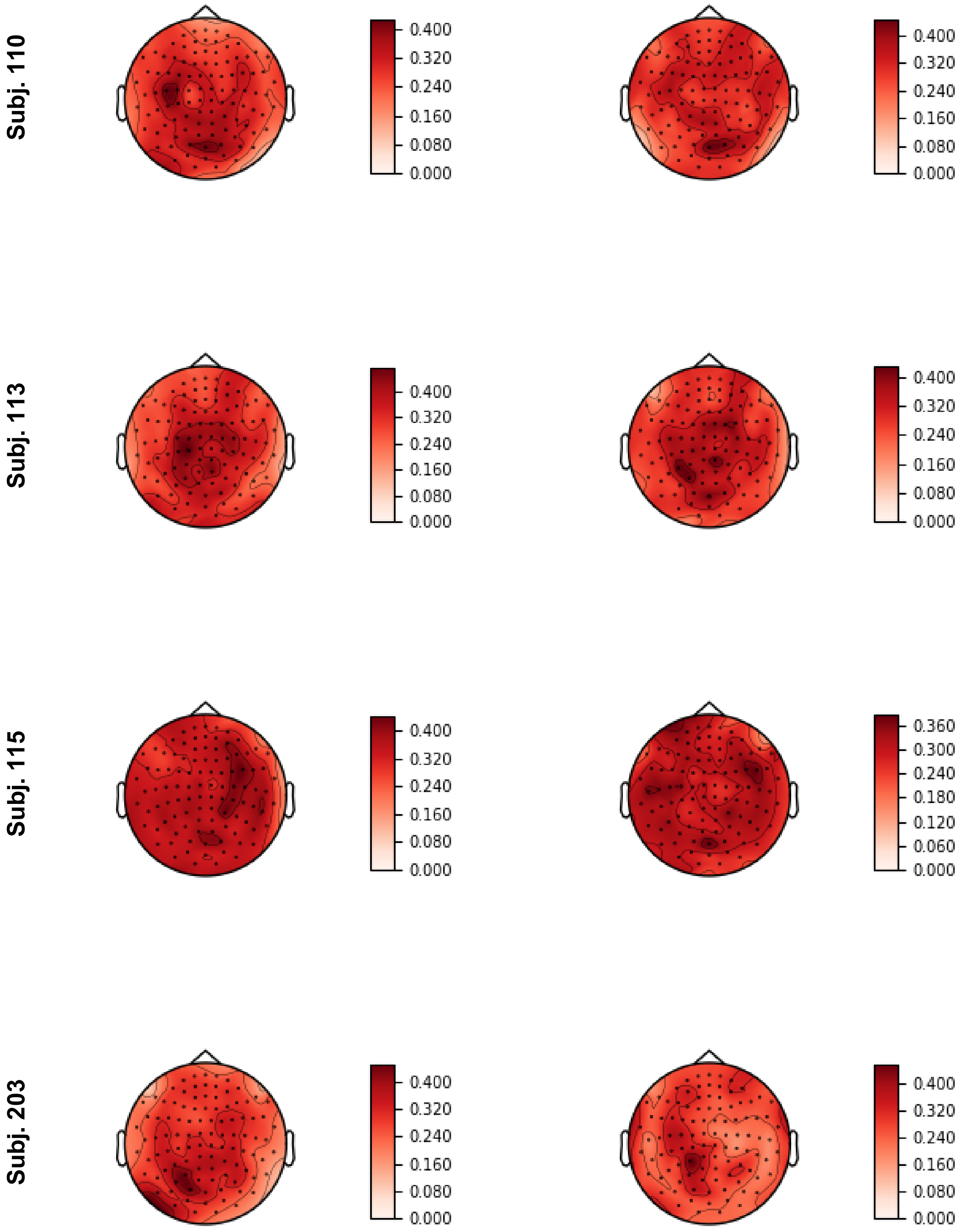
Subj. 110
0.400
0.320
0.240
0.160
0.080
0.000
0.400
0.320
0.240
0.160
0.080
0.000
Subj. 113
0.400
0.320
0.240
0.160
0.080
0.000
0.400
0.320
0.240
0.160
0.080
0.000
Subj. 115
0.400
0.320
0.240
0.160
0.080
0.000
0.360
0.300
0.240
0.180
0.120
0.060
0.000
Subj. 203
0.400
0.320
0.240
0.160
0.080
0.000
0.400
0.320
0.240
0.160
0.080
0.000

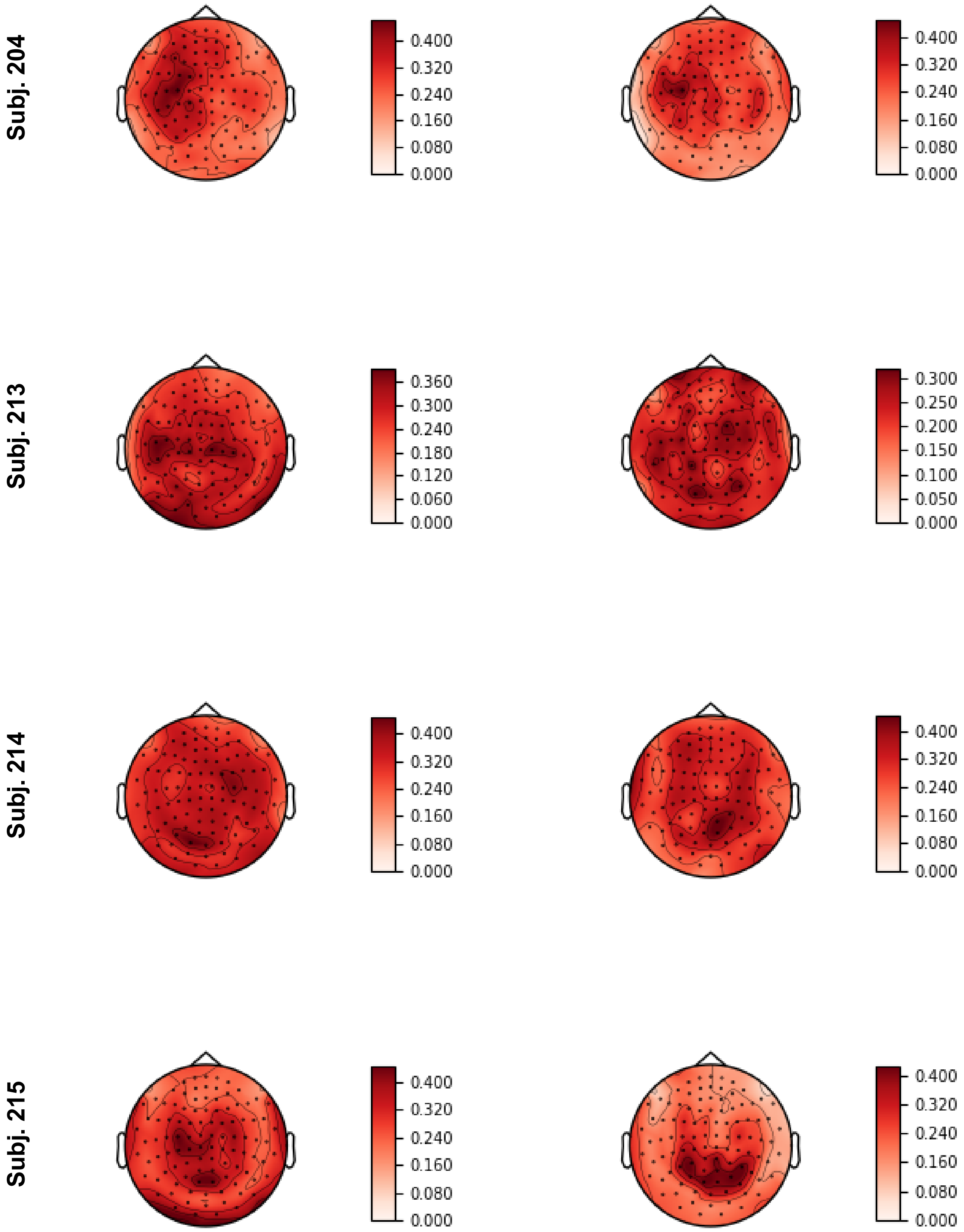
Subj. 204
0.400
0.320
0.240
0.160
0.080
0.000
Subj. 213
0.360
0.300
0.240
0.180
0.120
0.060
0.000
0.300
0.250
0.200
0.150
0.100
0.050
0.000
Subj. 214
Subj. 215

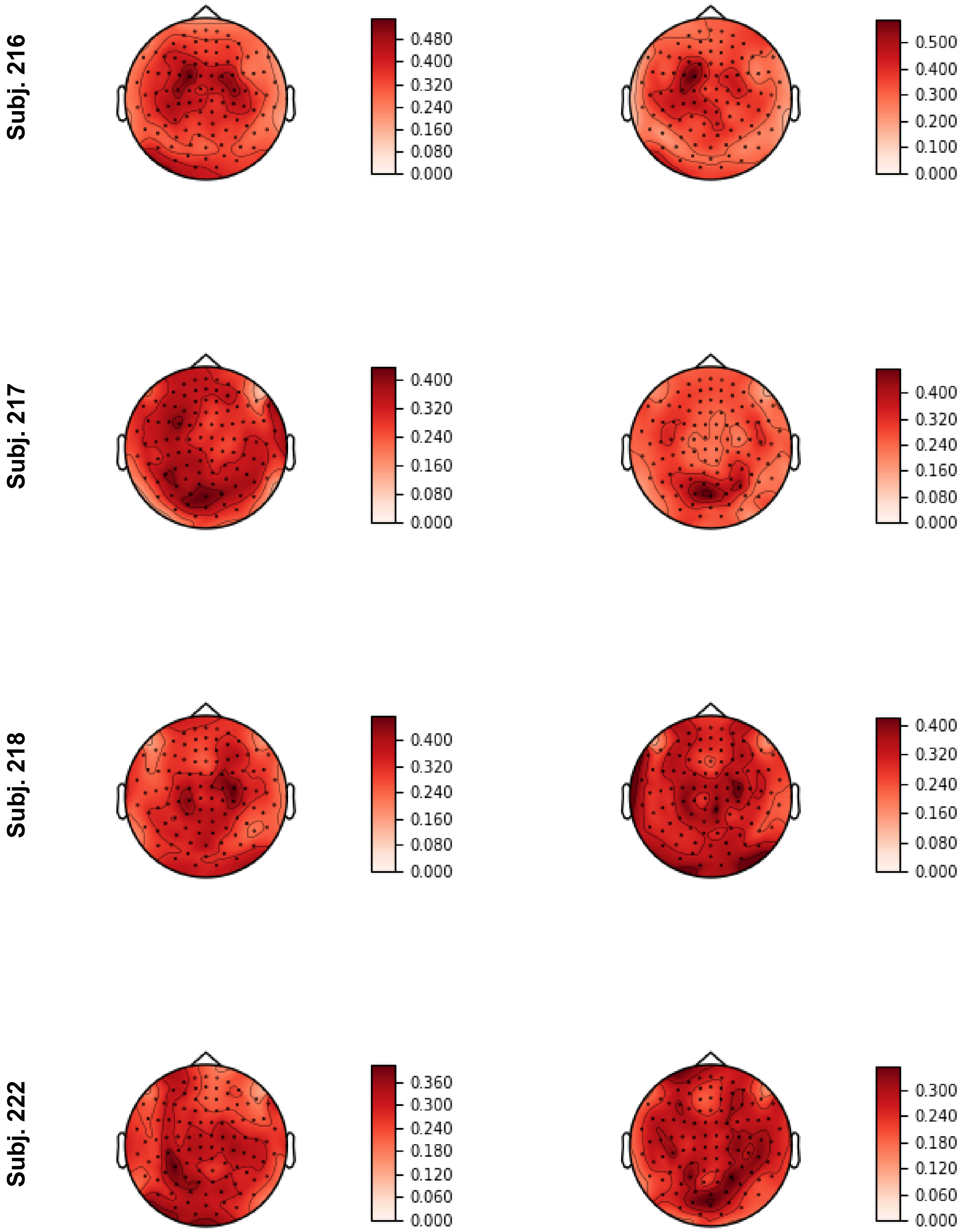
Subj. 216
0.480
0.400
0.320
0.240
0.160
0.080
0.000
0.500
0.400
0.300
0.200
0.100
0.000
Subj. 217
0.400
0.320
0.240
0.160
0.080
0.000
0.400
0.320
0.240
0.160
0.080
0.000
Subj. 218
0.400
0.320
0.240
0.160
0.080
0.000
0.400
0.320
0.240
0.160
0.080
0.000
Subj. 222
0.360
0.300
0.240
0.180
0.120
0.060
0.000
0.300
0.240
0.180
0.120
0.060
0.000

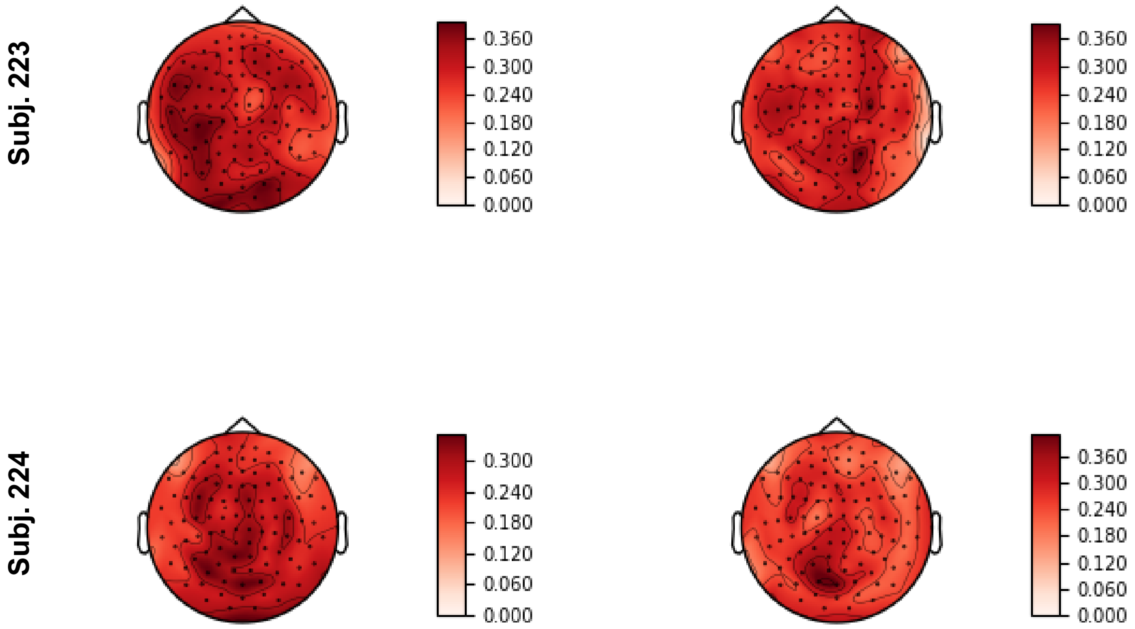

Supplementary Figure 3. Topographical maps of full explained variance fraction (FVE) for the participants which were not shown in Figure 4 in the main text.
